# Hippocampal Astrocytes Impact Postnatal Development of Inhibitory Connections, Parvalbumin Levels, Social, and Spatial Navigation Behaviors in a Mouse Model of Fragile X Syndrome

**DOI:** 10.1111/jnc.70541

**Published:** 2026-08-23

**Authors:** Victoria A. Wagner, Sarah Maples, Ritika Thapa, Jordan Wimberly, Ellie Pettijohn, Alexandra Varallo, Maham Rais, Samantha Sutley‐Koury, Peter Hickmott, Iryna M. Ethell

**Affiliations:** ^1^ Division of Biomedical Sciences and Biomedical Sciences Graduate Program University of California Riverside School of Medicine Riverside California USA; ^2^ Neuroscience Graduate Program University of California Riverside Riverside California USA; ^3^ Department of Psychology University of California Riverside Riverside California USA

**Keywords:** astrocytes, autism, Fragile X syndrome, GABA transport, hippocampal circuits, neurodevelopmental disorders, parvalbumin inhibitory interneurons, social behavior, spatial navigation, tonic inhibition

## Abstract

Fragile X Syndrome (FXS) is a leading genetic cause of autism‐like symptoms and intellectual disability, resulting from epigenetic silencing of the *Fragile X messenger ribonucleoprotein (Fmr1) gene*. Recent observations in FXS models suggest abnormal GABAergic signaling and excitation/inhibition imbalance may underlie the pathophysiology of FXS. As most studies have focused on neuronal mechanisms, the role of astrocytes in mediating defective inhibition in FXS is largely unknown. Our previous study showed the effects of astrocyte‐specific *Fmr1* conditional knockout (cKO) on cortical inhibitory circuit development using EEGs that were attributed to excess GABA synthesis by *Fmr1* KO astrocytes. As the hippocampus plays an important role in spatial learning and social behaviors that are altered in FXS, in this study we focused on dissecting the mechanism of abnormal inhibition in the CA1 hippocampus using slice electrophysiology. While we observed a reduction in the expression of synaptic GABA_A_ receptor subunits and overall density of perisomatic GABAergic synapses in cKO, the amplitude of spontaneous inhibitory postsynaptic currents (sIPSCs) was enhanced in pyramidal cells. In contrast to changes in phasic inhibition, astrocyte‐specific cKO did not affect tonic inhibition in pyramidal cells or the expression of extrasynaptic GABA_A_ receptors, which were both impaired in global KO. Our study suggests that elevated levels of extracellular GABA due to abnormal GABA transport in cKO astrocytes may contribute to the enhanced power of sIPSCs and potentially affect parvalbumin (PV) cell activity. Acute inhibition of GABA transport in astrocytes enhanced PV expression and improved spatial memory and socialization in cKO mice. Our work supports astrocytes as key players in the development and regulation of hippocampal inhibitory circuits in FXS, potentially through GABA transport.

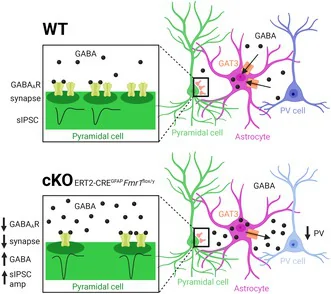

## Graphical abstract

Astrocyte‐specific postnatal deletion of *Fmr1* affects GABAergic gene expression and phasic inhibition of CA1 neurons in the hippocampus. While GABA transport in astrocytes does not alter tonic inhibition of CA1 pyramidal cells, it affects PV levels and is implicated in impaired spatial memories and social behaviors in astrocyte‐specific *Fmr1* cKO mice.

**Abbreviations**
BIC: Bicuculline
cKO: conditional knockout
Ctrl WT: ERT2‐Cre^*GFAP*^wild‐type
DEG: differentially expressed genes
DGGCs: dentate gyrus granule cells
E/I: excitation/inhibition
EEG: electroencephalogram
FMRP: Fragile X Messenger RibonucleoProtein
FXS: Fragile X Syndrome
GABA: gamma‐aminobutyric acid
GAT: GABA transporter 3
GO: Gene Ontology
HP: Hippocampus
HPLC: High‐Performance Liquid Chromatography
IP: intraperitoneally
KO: knockout
P: postnatal day
PC: pyramidal cell
PPI: paired pulse inhibition
PV: Parvalbumin
sIPSC: Spontaneous Inhibitory Postsynaptic Current
SNAP‐5114: SNAP
SP: Stratum Pyramidal
WT: wild type

## 1 Introduction

Fragile X Syndrome (FXS) is the most common genetic form of autism spectrum disorders (ASD) (Crawford et al. 2001). FXS occurs due to CGG repeat expansion in the *Fragile X messenger ribonucleoprotein 1* (*Fmr1*) *gene* that results in hypermethylation and subsequent loss of the gene product Fragile X Messenger RibonucleoProtein (FMRP) (Verkerk et al. 1991). FMRP is a translational regulator of hundreds of genes and can directly bind mRNAs as well as indirectly coordinate developmental gene cascades through regulation of microRNA expression (Darnell et al. 2011; Eberhart et al. 1996; Jin et al. 2004; Laggerbauer et al. 2001; Zalfa et al. 2007). FMRP loss results in altered and excessive protein synthesis, resulting in behavioral symptoms including intellectual disability, developmental delays, repetitive behaviors, social communication deficits, and sometimes seizures (Bailey Jr et al. 2008; Kau et al. 2004; Musumeci et al. 1999), potentially due to an imbalance of excitation/inhibition (E/I) during neural circuit development (Banker et al. 2021; Papatheodoropoulos 2025).

A non‐human model of FXS, the *Fmr1* knockout (KO) mouse, supports mechanistic study of circuit dysregulation. Increased excitability has been observed in the hippocampus of the *Fmr1* KO mice (Booker et al. 2019; Chuang et al. 2005; Deng et al. 2019; Luque et al. 2017), including elevated baseline gamma power observed with electroencephalogram (EEG) (Sinclair et al. 2017). E/I imbalance in FXS is thought to emerge from altered and uncoordinated inhibition that fails to regulate excitation (Nomura 2021; Paluszkiewicz et al. 2011). Indeed, inhibitory deficits are observed in *Fmr1* KO mice and human individuals with FXS, including the reduction of GABA_A_ receptor (GABA_A_R) expression (Curia et al. 2009; D'Hulst et al. 2006). Lower GABA_A_R expression was reported in both cortex and the hippocampus (Deng et al. 2022; El Idrissi et al. 2005; Sabanov et al. 2017) and was suggested to underlie reduced tonic inhibition (Curia et al. 2009; Modgil et al. 2019). Additionally, alterations in spontaneous IPSC (sIPSC) frequency and amplitude and paired pulse inhibition (PPI) have also been observed in excitatory neurons, either increasing or decreasing depending on the brain area (Cea‐Del Rio et al. 2020; Centonze et al. 2008; Leontiadis et al. 2023; Sabanov et al. 2017). While most phenotypic features in FXS have been attributed to neuronal changes, the involvement of astrocytes in the abnormal inhibition in FXS is still unclear.

FXS astrocytes have been shown to induce an FXS phenotype in wild‐type (WT) neurons in mouse hippocampal primary cell cultures and human differentiated cortical cell cultures (Jacobs and Doering 2010; Pacey and Doering 2007; Sharma et al. 2023). The role of astrocytes in FXS has primarily focused on facets of excitation (Bergdolt et al. 2026; Higashimori et al. 2016); however, astrocytes are known to modulate tonic and phasic inhibition through the transport and metabolism of GABA (Héja et al. 2012; Kwak et al. 2020; Lalo et al. 2014; Matos et al. 2018; Park et al. 2025). Recent work from our lab has revealed that human astrocytes from FXS individuals and *Fmr1* KO mouse astrocytes produce excess GABA that affects cortical inhibition, resulting in sensory processing deficits and hyperactivity (Wagner et al. 2026).

Taking into account the role of the hippocampus in spatial learning and social behaviors that are altered in FXS (Hitti and Siegelbaum 2014), the goal of this study was to determine whether astrocyte‐specific deletion of *Fmr1* during the developmental period of hippocampal inhibitory circuit refinement would affect phasic and tonic GABAergic transmission in the hippocampus. To address this goal, we used transcriptional, cellular, molecular, electrophysiological, and behavioral approaches to investigate astrocyte‐specific mechanisms of inhibitory circuit dysregulation in the hippocampus. We examined both conditional and global *Fmr1* KO models using hippocampal slice electrophysiology to further elucidate and validate the contributions of astrocytes to FXS pathology. Our results demonstrate a compelling role for astrocytic GABA transport in the regulation of inhibition in the hippocampus and reveal novel insights into a non‐neuronal mechanism of FXS.

## 2 Materials and Methods

### 2.1 Mice

To achieve specific *Fmr1* deletion in astrocytes, two mouse lines were generated. In Group 1, ERT2‐Cre^*GFAP*^ (B6.Cg‐Tg(*GFAP*‐cre/ERT2)505Fmv/J RRID: IMSR_JAX:012849) male mice were crossed with *Fmr1* ^flox/flox^ female mice (generated in and obtained from the laboratory of Dr. David Nelson) (Baylor College of Medicine, Houston, Texas87) to obtain ERT2‐Cre^*GFAP*^ *Fmr1* ^flox/y^ condition KO (cKO) male mice. ERT2‐Cre^*GFAP*^ mice were designated as control WT (Ctrl WT). In Group 2, ERT2‐Cre*GFAP* mice and ERT2‐Cre*^GFAP^Fmr1* ^f^ ^lox/flox^ mice were crossed with Rosa‐CAG‐LSL‐tdTomato reporter mice (CAG‐tdTomato; RRID:IMSR_JAX:007909) to generate tdTomatoERT2‐Cre*^GFAP^* Ctrl WT and tdTomatoERT2‐Cre*^GFAP^Fmr1* ^flox/y^ cKO mice, respectively, allowing for tdTomato expression in astrocytes following tamoxifen injection and analysis of *Fmr1* levels. We also used global *Fmr1* KO mice on C57BL/6 background (Jackson Laboratories, JAX:003025) and their WT counterparts (Jackson Laboratories, JAX: 000664). Real‐time PCR‐based analysis of genomic DNA isolated from mouse tails was used to confirm genotypes by Transnetyx.

**FIGURE 1:**
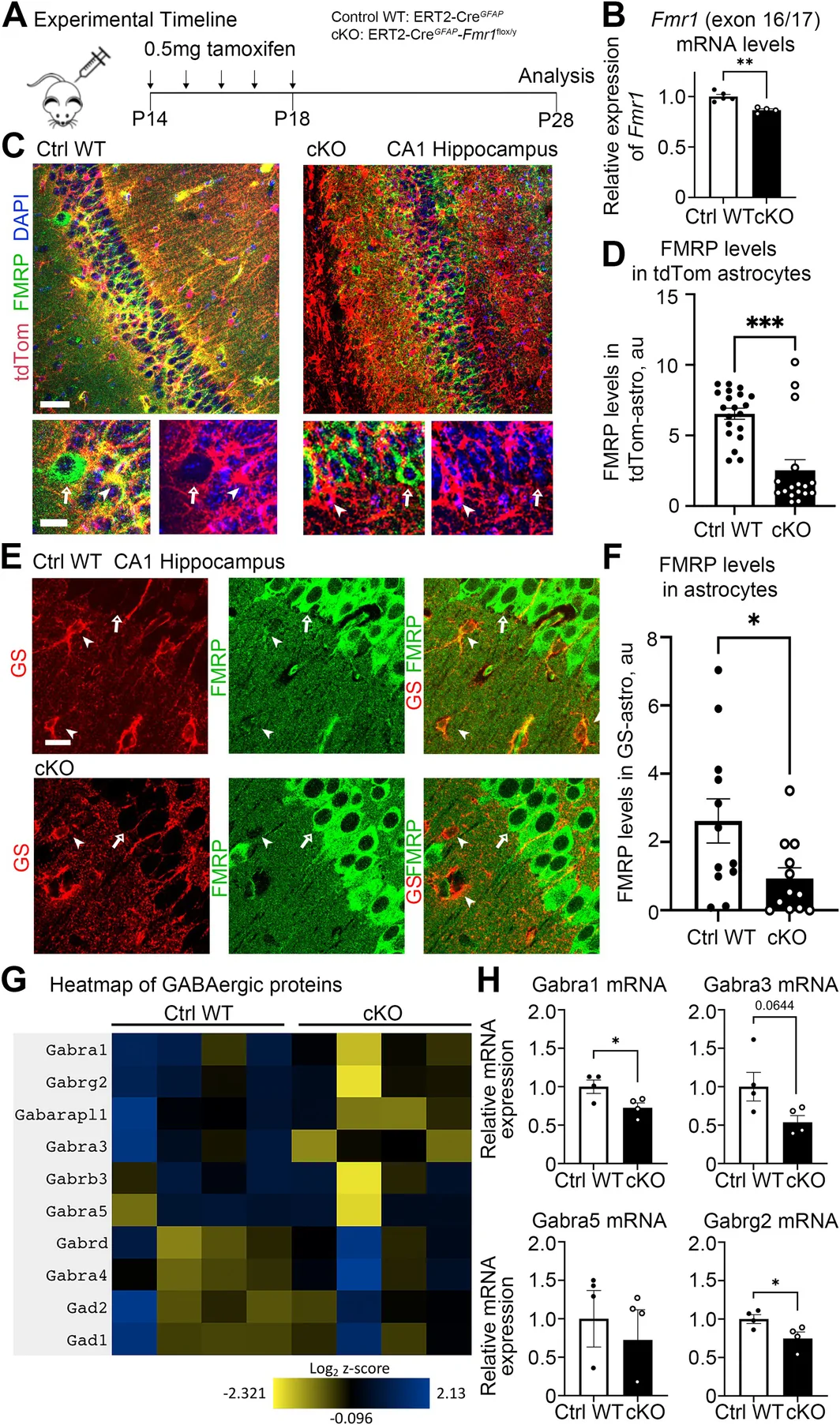
Astrocyte‐specific deletion of *Fmr1* during the P14–P28 developmental period results in significant changes in genes encoding GABA_A_ receptors in the hippocampus. (A) Graphics showing experimental timeline of *Fmr1* deletion in astrocytes. To achieve FMRP deletion in astrocytes, tamoxifen (0.5 mg) was intraperitoneally (IP) injected at P14 for 5 days; experiments were performed at postnatal day (P)28. The transgenic mouse groups used in this study were ERT2‐Cre^*GFAP*^control (Ctrl WT) and ERT2‐Cre^*GFAP*^ *Fmr1* ^flox/y^ condition KO (cKO). (B) Analysis of *Fmr1* (exon 16/17) mRNA levels in the hippocampus (HP) of Ctrl WT and cKO mice at P28 with qRT‐PCR. Graphs show mean ± SEM (*n* = 4–5 mice/group; **p < 0.01; *t*(7) = 5.024, two‐tailed unpaired *t*‐test, Table S1). *Fmr1* mRNA levels were significantly reduced in HP of cKO mice compared to Ctrl WT. (C) Confocal images showing tdTomato (tdTom)‐labeled Cre‐rexpressing astrocytes (red) and FMRP immunoreactivity (green) in the CA1 HP of P28 Ctrl WT (left panel) and cKO (right panel) mice. Scale bars, 50 μm (upper) and 25 μm (lower). (D) Quantitative analysis of FMRP levels in td‐Tom astrocytes in the CA1 HP at P28. Graphs show mean ± SEM (*n* = 17–20 images/4 mice per group; ***p = 0.0001, Mann–Whitney *U* test, Table S1). FMRP levels in td‐Tom astrocytes were significantly reduced in cKO mice compared to WT. (E) Confocal images showing glutamine synthetase (GS)‐labeled astrocytes (red) and FMRP immunoreactivity (green), where overlay appears yellow, in neurons (arrow) and astrocytes (arrowhead) in the CA1 HP of P28 Ctrl WT (upper panels) and cKO (lower panels) mice. Scale bar, 15 μm. (F) Quantitative analysis of FMRP levels in astrocytes in the CA1 HP at P28. Graph shows mean ± SEM (*n* = 12 images/3 mice per group; *p = 0.0325; Mann–Whitney Test, Table S1). FMRP levels in astrocytes were significantly reduced in cKO mice compared to controls. (G) Heatmap of genes from the Glial Profiling NanoString Panel encoding GABAergic proteins in the HP of cKO and Ctrl WT mice. Heatmap shows normalized gene expression data from low expression (yellow) to high expression (blue) and is generated using nSolver software. (H) Quantitative analysis of mRNA levels of GABA_A_ receptor subunits performed with Nanostring. Plots show mean ± SEM (*n* = 4 mice/group; *p < 0.05; *t*(6) = 2.519 for *Gabra1 gene*, *t*(6) = 2.262 for *Gabra3 gene*, t(6) = 0.5109 for *Gabra5 gene*, *t*(6) = 2.469 for *Gabrg2 gene*; two‐tailed *t*‐test, Table S1). We observed a significant decrease in GABA_A_ receptor subunits *Gabra1* and *Gabrg2* and a trend towards significance for *Gabra3* in the HP of cKO mice compared to Ctrl WT.

Ctrl WT and cKO mice received tamoxifen at P14 intraperitoneally (0.5 mg in 5 mg/mL of 1:9 ethanol/sunflower seed oil solution) once a day for 5 consecutive days, and analysis was performed at P28 (see “Experimental Timeline” in Figure 1a). Mice were used for immunohistochemistry, Western blot, qRT‐PCR, and electrophysiology. We also used global *Fmr1* KO mice for electrophysiology. Litters were randomly selected for vehicle or SNAP treatment. Although our study does not show direct pharmacokinetic data to confirm brain penetration, we utilized published data on SNAP farmacokinetics to determine dose and time point (Nakada et al. 2013; Mortensen et al. 2026; Lie et al. 2018). For all behavior tests, we used vehicle injected group as a control for SNAP injected group to consider the effects of injection.

Due to the small litter size, male offspring from each litter were assigned to specific experiments prior to inclusion of the next litter, allowing randomization across experimental groups while minimizing litter effects.

Regarding the use of only males in this study, we utilized breeding methods where we were breeding heterozygous females, allowing us to have both WT and cKO males as littermates. This breeding strategy left us with het females, no WT females, and only a few cKO females. Furthermore, potential effects of tamoxifen on ER‐dependent female behaviors made us shift the focus to males only in this study. Finally, the manifestation of FXS‐associated phenotypes is stronger in males as FXS is an X‐linked disorder, allowing consistent genotype‐dependent analysis. It is also consistent with standard practice in preclinical Fragile X research. However, future studies with females will allow separation of sex‐dependent mechanisms.

### 2.2 Methods Overview

As *Fmr1* is expressed in astrocytes during postnatal development (Jacobs and Doering 2010; Pacey and Doering 2007) and we investigated changes in gene expression during postnatal day (P)14–P21, we used ERT2‐Cre^*GFAP*^ line to delete *Fmr1* from astrocytes during the P14–P21 period by administering tamoxifen daily from P14 to P18 under isoflurane anesthesia. For isoflurane anesthesia, a controlled open‐drop method was administered under a chemical fume hood to minimize anesthetic exposure. We utilized a Cre‐dependent tdTomato reporter line to validate the astrocyte‐specific Cre expression and evaluated *Fmr1* deletion using immunolabeling against FMRP. For further confirmation of deletion of FMRP in astrocytes, we examined the expression of FMRP in the hippocampus of P28 mice using immunostaining. Once deletion was established, immunohistochemistry, Western blot, analysis of gene expression, and behavior were performed in P28 Ctrl WT and cKO male mice, and slice electrophysiology in P21–P28 WT, global KO, Ctrl WT, and cKO male and female mice.

At P28, we analyzed the effects of astrocyte‐specific *Fmr1* deletion on gene expression in the hippocampus using the NanoString “Glial Profiling Panel” and qRT‐PCR. Biochemical measurements of GABA_A_ receptors and GAT3 were done in the hippocampus of cKO and Ctrl WT mice using Western blot. Lastly, immunohistochemistry was used (1) to assess GABA levels in astrocytes; (2) to determine PV levels; and (3) to analyze inhibitory synapses by measuring vGAT/Gephyrin co‐localization in the hippocampus of cKO and Ctrl WT mice.

Electrophysiological recordings were performed in hippocampal slices from Ctrl WT, cKO, WT, and global *Fmr1* KO mice to determine spontaneous inhibitory postsynaptic currents (IPSCs) and tonic GABA currents in CA1 pyramidal cells in the presence of absence of SNAP‐5114 treatment to block astrocyte GABA transporter GAT3.

### 2.3 Immunofluorescence

Four hours following injection of either SNAP‐5114 (25 mg/mL) or vehicle (10% DMSO in saline, 10 mL/kg) IP injection, P28 male Ctrl WT and cKO mice were euthanized with isoflurane and perfused transcardially first with cold phosphate‐buffered saline (PBS, 0.1 M) to clear out the blood and then with 4% paraformaldehyde (PFA) in 0.1 M PBS for fixation. For isoflurane anesthesia, a controlled open‐drop method was administered under a chemical fume hood to minimize anesthetic exposure. Brains were removed and post‐fixed for 2 h in 4% PFA. 100 μm brain slices were obtained using a vibratome (5100 mz Campden Instruments). Hippocampus (HP) was identified using the brain atlas (Paxinos and Franklin 2004). For each brain, an average of 8 brain slices were collected.

Immunostaining for FMRP was performed using antigen retrieval methods, as previously described (Hooper et al. 2021). Briefly, 100 μm brain slices from P28 Ctrl WT and cKO male mice (with or without tdTomato expression) were stained overnight with mouse anti‐FMRP (1:200; Developmental Studies Hybridoma Bank, Cat# 7 g1‐1, RRID:AB_528251) with or without rabbit anti‐Glutamine Synthetase antibody (1:500, Sigma‐Aldrich Cat# G2781, RRID:AB_259853). Secondary antibodies were AlexaFluor‐488‐conjugated donkey anti‐mouse IgG (4 μg/mL, Invitrogen, catalog # A‐21202, RRID:AB_141607) with or without donkey anti‐rabbit Alexa Fluor 594 (4 μg/mL, Invitrogen, catalog #A‐21207, RRID:AB_141637). Slices were mounted on slides with Vectashield mounting medium containing DAPI (Vector Laboratories, catalog # H‐1200, RRID:AB_2336790) and Cytoseal.

Immunostaining for synaptic proteins and PV was performed in 100 μm brain slices containing hippocampus as previously described (Reinhard et al. 2019; Nguyen, Sutley, et al. 2020; Kulinich et al. 2020; Sutley‐Koury, Taitano‐Johnson, et al. 2024; Norman et al. 2025). Inhibitory presynaptic sites were detected by immunolabeling against vesicular GABA transporter (vGAT) using rabbit anti‐vGAT antibody (1:100, Synaptic Systems, catalog # 131002, RRID:AB_887871). Inhibitory postsynaptic sites were detected by immunolabeling against gephyrin using mouse anti‐gephyrin antibody (1:500, Synaptic Systems, catalog # 147111, RRID:AB_887719). Parvalbumin cells were detected by immunolabeling against parvalbumin using mouse anti‐parvalbumin antibody (Millipore Cat# MAB1572, RRID:AB_2174013). Secondary antibodies used were as follows: FITC‐conjugated goat anti‐mouse IgG (4 μg/mL, Invitrogen, catalog # 31569, RRID:AB_228306) and donkey anti‐rabbit Alexa Fluor 594 (4 μg/mL, Invitrogen, catalog #A‐21207, RRID:AB_141637). Slices were mounted on slides with Vectashield mounting medium containing DAPI (Vector Laboratories, catalog # H‐1200, RRID:AB_2336790) and Cytoseal.

Immunostaining for GABA and Glutamine Synthetase (GS) was performed in 100 μm brain slices containing hippocampus as previously described with modifications (Yoon et al. 2011; Ormel et al. 2020). For GABA, immunolabeling with guinea pig anti‐GABA antibody (1:1000, Abcam, catalog # ab17413, RRID:AB_443865) and rabbit anti‐GS (1:1000, Sigma‐Aldrich Cat# G2781, RRID:AB_259853, RRID:AB_141637) was done for 2 h in blocking buffer overnight at 4°C. For secondary antibody incubation with goat anti‐guinea pig Alexa Fluor 488 (4 μg/mL, Invitrogen, catalog #A‐11073, RRID:AB_2534117) and donkey anti‐rabbit Alexa Fluor 594 (4 μg/mL, Invitrogen, catalog #A‐21207) was done at room temperature. Slices were mounted on slides with Vectashield mounting medium containing DAPI (Vector Laboratories, #H1200) and sealed with Cytoseal. We have previously performed HPLC using FXS patient‐derived induced pluripotent stem cells differentiated into astrocytes in vitro, which showed increased GABA levels in cultured FXS astrocytes (Wagner et al. 2026). Performing HPLC in mouse brain tissue would mask the astrocyte‐dependent changes in extracellular GABA since the majority of the GABA comes from neurons, making the interpretation of the results difficult. The staining protocol was identical among groups, processing was done in mixed groups, and analysis was done separately by a different experimenter to minimize any bias.

### 2.4 Image Analysis

Confocal images of coronal brain slices containing CA1 HP were taken with an SP5 confocal laser‐scanning microscope (Leica Microsystems) as previously described with modifications (Koeppen et al. 2018; Nguyen, Koeppen, et al. 2020; Sutley‐Koury, Taitano‐Johnson, et al. 2024). Briefly, high‐resolution optical sections (1024 × 1024 pixels format) were captured with a 40× (1.2 NA) and 1× zoom at a 1 μm step interval to assess FMRP immunoreactivity. Confocal images of synaptic puncta were taken using a 40× objective (1.2 NA) and 1× zoom at high resolution (1024 × 1024 pixel format) with a 0.5 μm step interval. All images were acquired under identical conditions and processed for analysis as follows: (1) For analysis of overall FMRP immunoreactivity, CA1 hippocampal area was analyzed in each brain slice from at least 3 animals per group. Astrocytes were visualized with tdTomato or GS. Cell areas were outlined in ImageJ (RRID:SCR_002285) using selection tool, then cell area, integrated fluorescent intensity, and mean intensity were measured for FMRP in each astrocyte. (2) For analysis of vGAT/Gephyrin colocalization, three‐dimensional fluorescent images were created by the projection of each z stack containing 5–10 high‐resolution optical serial sections (1024 × 1024 pixels format) taken at a 0.5 μm interval in the X‐Y plane. vGAT puncta over 0.15 μm^3^ in size were rendered, followed by rendering gephyrin puncta over 0.11 μm^3^ in size that were within 1 μm of vGAT puncta using Neuroleucida 360 software (MicroBrightField RRID:SCR_001775). Puncta renderings were analyzed using Neurolucida Explorer software (MBF Bioscience, RRID:SCR_017348) which measured total number of puncta and the number of puncta with at least 10% colocalization. Total and colocalized puncta counts were normalized to the 3D volume. (3) For analysis of PV levels, z stacks containing high‐resolution optical serial sections (1024 × 1024 pixels format) taken at a 1 μm interval were compressed into average projections, auto‐adjusted, background subtracted, thresholded using isodata, converted to a binary image, despeckled, and watershed on ImageJ. Analyze particles was used to generate ROIs of PV positive cells and ROIs detecting artifacts were manually removed. Remaining ROIs were counted and normalized to area for density measurements and were applied to a max projection for measurement of mean intensity per cell. To transparently present the biological variability we reported mean in individual cells and images rather than averaging per mouse, that would otherwise be masked. This includes differences in GS‐positive astrocytic FMRP and GABA levels (natural variations in FMRP and GABA levels in astrocytes following conditional deletion), variability in synaptic staining across hippocampal sections (anterior–posterior axis), and natural variations in PV levels taking into account both low PV and high PV expressing cells. We transparently show cell‐to‐cell and section‐to‐section differences across groups, which is a common approach in this kind of analysis in the field (Rais et al. 2022; Wagner et al. 2026).

Statistical analysis was performed with appropriate parametric or non‐parametric tests depending on the normality test (Shapiro–Wilk) results using GraphPad Prism 10 software (RRID:SCR_002798). Statistical analysis was performed with an unpaired Student's two‐tailed *t*‐test if data passed the normality test. Data not passing the normality test used either the Kruskal–Wallis test or a non‐parametric test. All the *p*‐values for the normality test as well as the non‐parametric tests have been included in the Supporting Information. Data represent mean ± standard error of mean (SEM) for bar graphs, and median with quartiles for box plots.

### 2.5 RNA Extraction

P28 male Ctrl WT and cKO mice were euthanized with isoflurane and the HP was removed from each mouse (*n* = 4–5 mice per group) and cooled in PBS. Total RNA was extracted from using the RNeasy kit (Qiagen, Valencia, CA, USA) method as previously with minor modifications (Sutley‐Koury, Anderson, et al. 2024). First‐strand complementary DNA (cDNA) was generated using the High‐Capacity cDNA Reverse Transcription Kit (Catalog #4368814, Applied Biosystems), and reverse transcription of total RNA (500 ng) was performed for 120 min at 37°C. All surfaces for tissue collection and processing were sanitized using 70% ethanol and then treated with an RNAse inhibitor (RNaseZap, Thermo Fisher Scientific, catalog # AM9780, Waltham, MA, USA) to maintain the integrity of isolated RNA. cDNA was used for NanoString analysis and qRT‐PCR.

### 2.6 NanoString nCounter Gene Expression Assays and Analysis

nCounter gene expression assays (NanoString Technologies) were performed for the Glial Profiling NanoString Panel. Briefly, panel codeset probes were hybridized with 150 ng of total RNA per hippocampus sample over 18 h at 65°C according to NanoString protocol. Hybridized RNA was then diluted in molecular grade water and loaded into nCounter SPRINT cartridge (NanoString), placed into nCounter SPRINT Profiler, and quantified. RNA‐conjugated probes were counted via NanoString Sprint Profiler technology.

#### 2.6.1 nSolver and Advanced Analysis

Results from the panel were normalized in nSolver following best practices and analyzed using nSolver and Advanced Analysis software according to previously published protocols (Bergersen et al. 2021; Danaher et al. 2017). nSolver‐generated heat maps were created using normalized data and agglomerative clustering, a bottom‐up form of hierarchical clustering (NanoString User Manual C0019‐08). For Advanced Analysis, normalized data was used (NanoString User Manual 10030‐03). Differential expression (DE) analysis was performed to identify specific targets that exhibit significantly increased or decreased expression compared to Ctrl WT values. Gene set analysis was run to determine the change in direction of regulation within each pre‐defined gene set relative to Ctrl WT. Global significance scores, a summary T‐statistic used to measure change (NanoString User Manual 10030‐03) were calculated, and the directed global significance scores were expressed via heatmap. Pathway analysis was also conducted to determine overall changes in pathways based on the first principal component of the targets within a pathway as annotated by NanoString (NanoString User Manual 10030‐03). Direction of pathway change (up‐ or downregulated) was determined by cross referencing the pathway score with the corresponding volcano plot for that pathway. Summary pathway score plot colors are based on calculated scores and are represented as downregulation (yellow) to upregulation (blue).

#### 2.6.2 Statistical Analyses

Normalized linear counts for all genes in the panel were used in fold change analysis of cKO genes compared to Ctrl WT. Differentially expressed genes (DEGs) were defined by a *p*‐value < 0.05. Statistical analysis was performed with appropriate parametric or non‐parametric test depending on the normality test (Shapiro–Wilk) results using GraphPad Prism 10 software (RRID:SCR_002798). Statistical analysis was performed with unpaired two‐tailed Student's *t*‐test if data passed normality test using GraphPad Prism 7 software (RRID: SCR_002798). Data represent mean ± SEM. Gene enrichment and functional annotation analyses of DEGs was performed using open‐source functional enrichment tool DAVID (https://david.ncifcrf.gov/home.jsp). All raw NanoString data are available at: https://www.dropbox.com/sh/19b420pr1nl1zlh/AAAJ_qe0uhyIjeNzFAwVECFsa?dl=0.

### 2.7 Quantitative Real Time PCR Analysis

qRT‐PCR was carried out using SsoAdvanced Universal SYBR Green Supermix (Biorad catalog # #1725271, Hercules, CA, USA) with the following primers for mouse genes: *Fmr1*, *PV*, and *Slc6a11* (see Table S1 for sequences of primers used for qRT‐PCR). *GAPDH* was selected as a housekeeping gene and no changes in its expression were found across genotype. Relative quantification using the delta–delta (2^−ΔΔCq^) method was used to compare changes in gene expression between cKO mice and Ctrl WT mice. Reactions were run in triplicate for each animal. The sample size was based on our previously published work on mRNA analysis (Rais et al. 2022; Sutley‐Koury, Anderson, et al. 2024; Sutley‐Koury, Taitano‐Johnson, et al. 2024; Norman et al. 2025). Since we saw significant changes with the number of mice we used, further mice were not included to avoid the use of mice that were not necessary, abiding by the IACUC rules of principle of reduction. Statistical analysis was performed with appropriate parametric or non‐parametric test depending on the normality test (Shapiro–Wilk) results using GraphPad Prism 10 software (RRID:SCR_002798). Statistical analysis was performed with unpaired two‐tailed Student's *t*‐test if data passed normality test using GraphPad Prism 7 software (RRID:SCR_002798). Data represent mean ± SEM.

### 2.8 Western Blot Analysis

P28 male Ctrl WT and cKO mice were euthanized with isoflurane and the HP was removed from each mouse (*n* = 3–4 mice per group), cooled in PBS, and homogenized in ice‐cold lysis buffer (50 mM Tris–HCl, pH 7.4, 150 mM NaCl, 5 mM EDTA, 0.05% Triton X‐100, and 1 mM PMSF) containing protease inhibitor cocktail (Sigma, catalog # P8340) and phosphatase inhibitor cocktail (Sigma, catalog # P0044). The samples were processed as previously described with modifications (Miura et al. 2001; Lovelace et al. 2020). The samples were rotated at 4°C for at least 30 min to allow for complete cell lysis and then cleared by centrifugation at 13200 rpm for 20 min at 4°C. Supernatants were isolated and boiled in reducing sample buffer (Laemmli 2× concentrate, catalog # S3401, Sigma), and separated on 8%–16% Tris‐Glycine SDS‐PAGE precast gels (catalog # EC6045BOX, Life Technologies). Proteins were transferred onto Protran 0.45 μm Nitrocellulose membrane (GE Healthcare catalog # 10600007, Chicago, Illinois, USA) and blocked for 1 h at room temperature in 5% skim milk (Catalog #170‐6404, Bio‐Rad). Primary antibody incubations were performed overnight at 4°C with antibodies diluted in TBS/0.1% Tween‐20/1% BSA. The following primary antibodies were: rabbit anti‐ GABA_A_Rγ2 (1:1000; ThermoFisher Scientific, catalog #14104‐1‐AP, RRID:AB_10693527); rabbit anti‐ GABA_A_Rα5 (1:1000; ThermoFisher Scientific, catalog #PA5‐31163, RRID:AB_2548637); rabbit anti‐GAT3 (1:1000; Millipore Cat# AB1574, RRID:AB_90779); and mouse anti‐βactin (1:1000; Santa Cruz Biotechnology Cat# sc‐47 778, RRID:AB_626632).

Blots were washed 3 × 10 min with TBS/0.1% Tween‐20 and incubated with the appropriate HRP‐conjugated secondary antibodies for 2 h at room temperature in a TBS/0.1% Tween‐20/1% BSA solution. The secondary antibodies used were HRP‐conjugated donkey anti‐mouse IgG (Jackson ImmunoResearch, catalog #715‐035‐150, RRID: AB_2340770) or HRP‐conjugated goat anti‐rabbit IgG (Jackson ImmunoResearch, catalog #111‐035‐003, RRID: AB_2313567). After secondary antibody incubations, blots were washed 3 × 10 min in TBS/0.1% Tween‐20, washed 1 × 10 min in TBS, incubated in Pierce ECL Western Blotting Substrate (Thermo Scientific, catalog #32106) and imaged using the ChemiDoc imaging system (Bio‐Rad, RRID:SCR_019037). For re‐probing, membrane blots were washed in stripping buffer (2% SDS, 100 mM β‐mercaptoethanol, 50 mM Tris‐HCl, pH 6.8) for 30 min at 55°C, then rinsed repeatedly with TBS/0.1% Tween‐20, finally blocked with 5% skim milk, and then re‐probed. The first blot was probed for GABRG2, GAT3, and β‐actin. The second blot was probed for GABRA5 and β‐actin. Band density was analyzed by measuring band and background intensity using Adobe Photoshop CS5.1 software (RRID:SCR_014199). 3–4 samples per group (Ctrl WT vs. cKO) were run per blot, and precision/tolerance (P/T) ratios for individual cKO samples were normalized to P/T ratios of Ctrl WT samples with similar actin levels. This western blot experiment was repeated with a different group of mice with similar results. The sample size was based on our previously published work on protein level analysis (Rais et al. 2022; Sutley‐Koury, Anderson, et al. 2024; Sutley‐Koury, Taitano‐Johnson, et al. 2024; Norman et al. 2025). Since we saw significant changes with the number of mice we used, further mice were not included to avoid the use of mice that were not necessary, abiding by the IACUC rules of principle of reduction. Statistical analysis was performed with appropriate parametric or non‐parametric test depending on the normality test (Shapiro–Wilk) results using GraphPad Prism 10 software (RRID:SCR_002798). Statistical analysis was performed with unpaired two‐tailed Student's *t*‐test if data passed normality test using GraphPad Prism 7 software (RRID: SCR_002798). Data represent mean ± SEM.

### 2.9 Electrophysiology

Animals aged P21–P28 from Ctrl WT, cKO, WT, and global *Fmr1* KO were anesthetized with isoflurane, brains were dissected, and 300 μm coronal sections were sliced in ice‐cold sucrose‐containing artificial cerebrospinal fluid (sACSF) containing (in mM) 2.5 CaCl2, 201.6 sucrose, 119 NaCl, 2.5 KCl, 1.3 MgSO4, 1 NaH2PO4, 26.2 NaHCO3, 11 D‐glucose, 3 kyneuric acid, 2 Na‐pyruvate, and 3.5 MOPS. Slices were incubated in sucrose ACSF for 30 min to acclimate to room temperature. Slices were then transferred to nifedipine‐enhanced ACSF containing (in mM) 2.5 CaCl2, 119 NaCl, 2.5 KCl, 1.3 MgSO4, 1 NaH2PO4, 26.2 NaHCO3, 11 d‐glucose, and 0.015 nifedipine and allowed to incubate for 45 min at room temperature. Slices were transferred to a recording chamber and allowed to equilibrate for at least 10 min while continuously perfused with oxygenated ACSF at a flow rate of 1 mL/min at 30.5°C.

Blind, whole‐cell patch–clamp recordings were obtained from CA1 pyramidal neurons using pipettes made from glass capillaries using a horizontal puller (P‐87, Sutter Instruments, Novato, CA, USA). Pipette resistance ranged from 3 to 9 mOhms using high‐cesium internal solution containing (in mM) 128 Cs‐gluconate, 7 CsCl, 0.010 QX‐314, 2 Mg2ATP, 0.3 Na2GTP, 7 KCl, 1 EGTA, 10 HEPES, and 0.2% biocytin for postlabeling and was pH adjusted to 7‐7.4. CA1 pyramidal neurons were voltage clamped and held at 0 mV. Holding the voltage at 0 mV and having high‐cesium internal solution allowed for efficient measurement of spontaneous IPSCs, alongside improving cell health and stability (Chen et al. 2025). Tonic inhibitory currents were recorded using an Axoclamp 2B amplifier (Axon Instruments), filtered through a Brownlee Precision Model 440 Instrumentation Amplifier, noise‐filtered by a Quest Scientific Hum Bug (50/60hz), transferred via a digitizer (National Instruments SCB‐68, 10 kHz), and acquired by Igor Pro 3.14 (Wave metrics Inc., Portland, OR) data acquisition system on a Macintosh G4. Access resistance was measured throughout recording and if it changed by > 20% the recording was rejected. After 8 min, once the cell stabilized, at least 75 s baseline data were recorded at room temperature to elongate the time of brain slice viability (Chen et al. 2025). Furthermore, since the same experimental condition was maintained for all experimental groups, temperature‐dependent effects can be eliminated. Then, a 100 μM bicuculline ACSF solution was introduced to the bath. At 5, 8, and 10 min after the bath change, 50–75 s current was recorded. The effects of bicuculline saturated at 10 min, so data from this timepoint were used for quantification of tonic GABA currents.

For experiments using SNAP‐5114, SNAP‐5114 was reconstituted as 25 mg/mL in DMSO and diluted to a working concentration of 50 μM SNAP in ACSF. For recording its effects on tonic inhibitory current, baseline was determined as above, then the 50 μM SNAP‐5114 was bath‐applied for 10 min and the resulting current was recorded for 50–75 s. SNAP‐5114 application was followed by application of 100 μM BMI plus 50 μM SNAP‐5114 in ACSF for 10 min, after which 50–75 s current was recorded. Note all drugs, unless otherwise noted, were purchased from Sigma Aldrich Inc., St. Louis, MO.

Current data were analyzed using Igor Pro 9.0 (Wave metrics Inc). The tonic current level was determined for baseline and after bath application of each drug by averaging 0.1 s of current from a range that had no spontaneous IPSCs. The magnitude of the tonic current was calculated by subtracting the baseline current level from the current level in the presence of BMI (Difference from Baseline). The effect of SNAP‐5114 was also determined by subtracting the current levels during SNAP‐5114 from the baseline current level. sIPSCs were detected based on amplitude > 1 pA above baseline and kinetics (rapid rise time, exponential fall). Amplitudes were measured from baseline. Events were analyzed for 20–50 events. Frequency was determined by dividing the number of events by the time interval analyzed. Statistical analysis was performed with appropriate parametric or non‐parametric test depending on the normality test (Shapiro–Wilk) results using GraphPad Prism 10 software (RRID:SCR_002798). For electrophysiology study only, we used both tamoxifen administered Ctrl WT and un‐injected WT and found not significant differences between groups and combined them to increase power for our statistical analysis (Table S3). Statistical significance was analyzed with unpaired two‐tailed Student's *t*‐test or one‐way ANOVA followed by Dunnett's post hoc test if data passed normality test using Prism 10 software (GraphPad Software, RRID:SCR_002798). Data represent mean ± SEM.

### 2.10 Social Novelty Test

Sociability and social memory were studied in male P27‐P29 Ctrl WT (*n* = 30), cKO (*n* = 43) mice, cKO vehicle mice (*n* = 14) treated with vehicle, and male cKO SNAP mice (*n* = 10) treated with SNAP‐5114 (25 mg/mL), with both treatments delivered by IP injection 4 h prior to the start of testing, using a three‐chamber test as described previously with minor modifications (Rais et al. 2022). Briefly, a rectangular box contained three adjacent chambers 19 × 45 cm each, with 30‐cm‐high walls and a bottom constructed from clear Plexiglas. The three chambers were separated by dividing walls, which were made from clear Plexiglas with openings between the middle chamber and each side chamber. Removable doors over these openings permitted chamber isolation or free access to all chambers. All testing was done in a brightly lit room (650 lx), between 9:00 A.M. and 2:00 P.M. Before testing, mice were housed in a room with a 12 h light/dark cycle with ad libitum access to food and water. The cages were transferred to the behavioral room 30 min before the first trial began for habituation. The test mouse was placed in the central chamber with no access to the left and right chambers and allowed to habituate to the test chamber for 5 min before testing began. Session 1 measured sociability. In Session 1, another mouse (Stranger 1) was placed in a wire cup‐like container in one of the side chambers. The opposite side had an empty cup of the same design. The doors between the chambers were removed, and the test mouse was allowed to explore all three chambers freely for 10 min, while being digitally recorded from above. The following parameters were monitored: the duration of direct contact between the test mouse and either the stranger mouse or empty cup and the duration of time spent in each chamber. Session 2 measured social memory and social novelty preference. In Session 2, a new mouse (Stranger 2) was placed in the empty wire cup in the second side chamber. Stranger 1, a now familiar mouse, remained in the first side chamber. The test mouse was allowed to freely explore all three chambers for another 10 min, while being recorded, and the same parameters were monitored. Placement of Stranger 1 in the left or right side of the chamber was randomly altered between trials. The floor of the chamber was cleaned with 2%–3% acetic acid, 70% ethanol, and water between tests to eliminate odor trails. Assessments of the digital recordings were performed blind to the condition, where the person conducting the analysis was blind to the genotype of the mouse in the behavioral video being analyzed. using TopScan Lite software (Clever Sys Inc., VA). To measure changes in social preference, percent time spent in each chamber was calculated during first 5 min in each test. The sample size was based on our previously published work on behavioral analysis (Rais et al. 2022; Sutley‐Koury, Anderson, et al. 2024; Sutley‐Koury, Taitano‐Johnson, et al. 2024; Norman et al. 2025). Since we saw significant changes with the number of mice we used, further mice were not included to avoid the use of mice that were not necessary, abiding by the IACUC rules of principle of reduction. Statistical analysis was performed with appropriate parametric or non‐parametric test depending on the normality test (Shapiro–Wilk) results using GraphPad Prism 10 software (RRID:SCR_002798). Statistical analysis for social preference and social novelty preference was performed using two‐way ANOVA with Šídák's multiple comparisons test using GraphPad Prism 10 software (RRID: SCR_002798). Data represent median and quartiles.

### 2.11 Object Location Memory Test

Spatial memory was studied in male P27‐P29 Ctrl WT (*n* = 8) and Ctrl cKO (*n* = 8), and cKO mice treated either with vehicle (*n* = 8) or SNAP (25 mg/mL; *n* = 13), with both treatments delivered by IP injection 4 h prior to the start of testing. The test was conducted using a 72 × 72‐cm open‐field arena with 50‐cm‐high walls constructed from opaque acrylic sheets with a clear acrylic sheet for the bottom. One side of the wall had a removable blue and white striped paper attached behind the opaque acrylic sheets to mark the wall as contextual cue. All testing was done in a brightly lit room (650 lx), between 9:00 A.M. and 2:00 P.M. Before testing, mice were housed in a room with a 12 h light/dark cycle with ad libitum access to food and water. The cages were transferred to the behavioral room 30 min before the first trial began for habituation. The floor of the arena was cleaned with 2%–3% acetic acid, 70% ethanol, and water between tests to eliminate odor trails. Two objects of different color, texture, and height were placed on two different locations on the same (contextual cue wall) side of the arena on the first day of the test. On acquisition phase, Day 1, the test mouse was placed in the corner of the arena containing Object 1 and allowed to explore for 10 min, while being digitally recorded from above. On Day 2, Object 2 was moved to the other side of the arena, away from the contextual wall. The mouse was placed in the corner containing Object 1, same as Day 1, and allowed to explore for 10 min, while being digitally recorded from above. The same objects were assigned as Object 1 and Object 2 for all mice. Analysis of the video was done blind, where the person conducting the analysis was blind to the genotype of the mouse in the behavioral video being analyzed using TopScan Lite software (Clever Sys Inc., VA). Object Preference Index was calculated for each object as follows $\frac{\text{Time spent with object}\;\mathrm{on}\;\mathrm{day}\;2}{\text{Time spent with object}\;\mathrm{on}\;\mathrm{day}\;1+\text{Time spend with object}\;\mathrm{on}\;\mathrm{day}\;2}$. Preference index > 0.5 indicates preference for the object on Day 2, while preference index less or equal to 0.5 indicates no preference. The sample size was based on our previously published work on behavioral analysis (Rais et al. 2022; Sutley‐Koury, Anderson, et al. 2024; Sutley‐Koury, Taitano‐Johnson, et al. 2024; Norman et al. 2025). Since we saw significant changes with the number of mice we used, further mice were not included to avoid the use of mice that were not necessary, abiding by the IACUC rules of principle of reduction. Statistical analysis was performed with appropriate parametric or non‐parametric test depending on the normality test (Shapiro–Wilk) results using GraphPad Prism 10 software (RRID:SCR_002798). Statistical analysis was performed using Tukey's multiple comparisons and Uncorrected Fisher's LSD post hoc test using GraphPad Prism 10 software (RRID: SCR_002798). Fisher's Least Significant Difference post hoc testing was used due to our limited number of group comparisons, where this test provides greater sensitivity, along with its high statistical power to detect true differences. Data represent median and quartiles.

## 3 Results

### 3.1 Astrocyte‐Specific *Fmr1* Deletion in Hippocampus During Postnatal Developmental Period Affects Expression of Genes Encoding GABA_A_ Receptors

To investigate the role of astrocytes in abnormal inhibition in FXS, we first investigated how early postnatal deletion of FMRP from astrocytes impacts expression of GABAergic genes. To achieve specific *Fmr1* deletion in astrocytes, ERT2‐Cre^*GFAP*^ *Fmr1* ^flox/y^ conditional KO mice (cKO) were generated, and ERT2‐Cre^*GFAP*^ wild‐type (Ctrl WT) mice were used as controls. Ctrl WT and cKO mice received tamoxifen at postnatal day (P)14 intraperitoneally (IP, 0.5 mg in 5 mg/mL of 1:9 ethanol/sunflower seed oil solution) once a day for 5 days, and analysis was performed at P28 (Figure 1A). All control and experimental groups receiving tamoxifen ruled out the possibility of non‐specific effects of tamoxifen administration. For randomization, since the WT and cKO were littermates, we injected all mice with tamoxifen at P14–P18 without knowing the genotype and genotype was determined later at P21. This allowed us to reduce littermate bias.

We first assessed how *Fmr1* expression was affected in hippocampus (HP) in our cKO model and found a small but significant reduction in *Fmr1* mRNA in HP of cKO compared to Ctrl WT (Figure 1B, Table S1). The use of an entire tissue indicates *Fmr1* is still being expressed in most cells (such as neurons) and that our conditional knockout had reduced *Fmr1* (in astrocytes) but not ablated overall expression, confirming cell‐specific deletion. In addition, Rosa‐CAG‐LSL‐tdTomato reporter mice were used to generate tdTomatoERT2‐Cre^*GFAP*^ *Fmr1* ^flox/y^ cKO mice and tdTomatoERT2‐Cre^*GFAP*^ Ctrl WT mice, allowing identification of Cre‐expressing cells that resembled astrocyte morphology (red, Figure 1C). There was a significant reduction of FMRP immunoreactivity (green) in tdTomato‐positive cells (red), suggesting the efficient knockout of FMRP from astrocytes (Figure 1C,D, Table S1). To address the possibility of a potential off‐target Cre expression in small numbers of neurons (~1% showing tdTomato expression), we also analyzed FMRP immunoreactivity in astrocytes immunolabeled against astrocyte marker Glutamine Synthetase (GS) in the CA1 HP of Ctrl WT and cKO mice at P28 (Figure 1E, Table S1). FMRP immunoreactivity was significantly decreased in CA1 astrocytes of cKO mice compared to Ctrl WT (Figure 1F, Table S1). Both results confirm successful deletion of FMRP specifically from developing astrocytes during the postnatal P14–P28 window using our model.

To determine if postnatal deletion of *Fmr1* from astrocytes affects expression of glial and neuronal genes associated with GABAergic transmission, we analyzed mRNA levels of selected genes in the hippocampus of Ctrl WT and cKO mice. Hippocampal mRNA expression was analyzed with the NanoString “Glial Profiling” panel. Analysis identified 81 differentially expressed genes (DEGs), including 66 downregulated and 15 upregulated DEGs (Table S1). Although a number of the 81 DEGs were glial‐specific (*Aqp4*, *S100a10*, *Aldh1a1*, and *Nes* were upregulated; and *Serpini1*, *Slc25a4*, *Serpina3n*, *Slc8a1*, *Ppp2r2b*, and *P2rx7* were downregulated), some DEGs were associated with synaptic signaling, regulation of synaptic plasticity, GABAergic synaptic transmission, and GABA receptor complex. Further Gene Ontology (GO) analysis indicated strong association of downregulated DEGs with the “Synaptic vesicle cycle” (*rab3a, cacna1b, rims1, snap25, syt1*, and *stxbp1*) and “GABAergic synapse” (*gnai1, cacna1b, gabra1, gabra3*, and *gabrg2*) pathways in the Kyoto Encyclopedia of Genes and Genomes (KEGG) pathway database (Figure S1). As previous studies have indicated that GABA_A_ receptors (GABA_A_R) levels are affected in FXS (Braat et al. 2015; D'Hulst et al. 2006; Gao et al. 2018; Gatto et al. 2014; Sabanov et al. 2017), we further analyzed the specific genes encoding subunits of GABA_A_R in the hippocampus.

NanoString normalized gene expression data indicate that some, but not all, genes associated with GABAergic transmission were dysregulated (Figure 1G). mRNA expression levels of *Gabra1*, *Gabra3*, and *Gabrg2* genes encoding synaptic GABA_A_R subunits were significantly decreased in cKO mice compared to Ctrl WT (Figure 1H, Table S1). Interestingly, we found no changes in the expression of the *Gabra5* gene that encodes the primarily extrasynaptic GABA_A_R subunit (Figure 1H, Table S1). Taken together, these data indicate that postnatal deletion of *Fmr1* from astrocytes affected the expression of synaptic GABA_A_R subunits, while the levels of extrasynaptic GABA_A_R subunits remained unchanged.

### 3.2 Density of Inhibitory Synapses Is Reduced in CA1 Hippocampus Following Astrocyte‐Specific *Fmr1* Deletion

**FIGURE 2:**
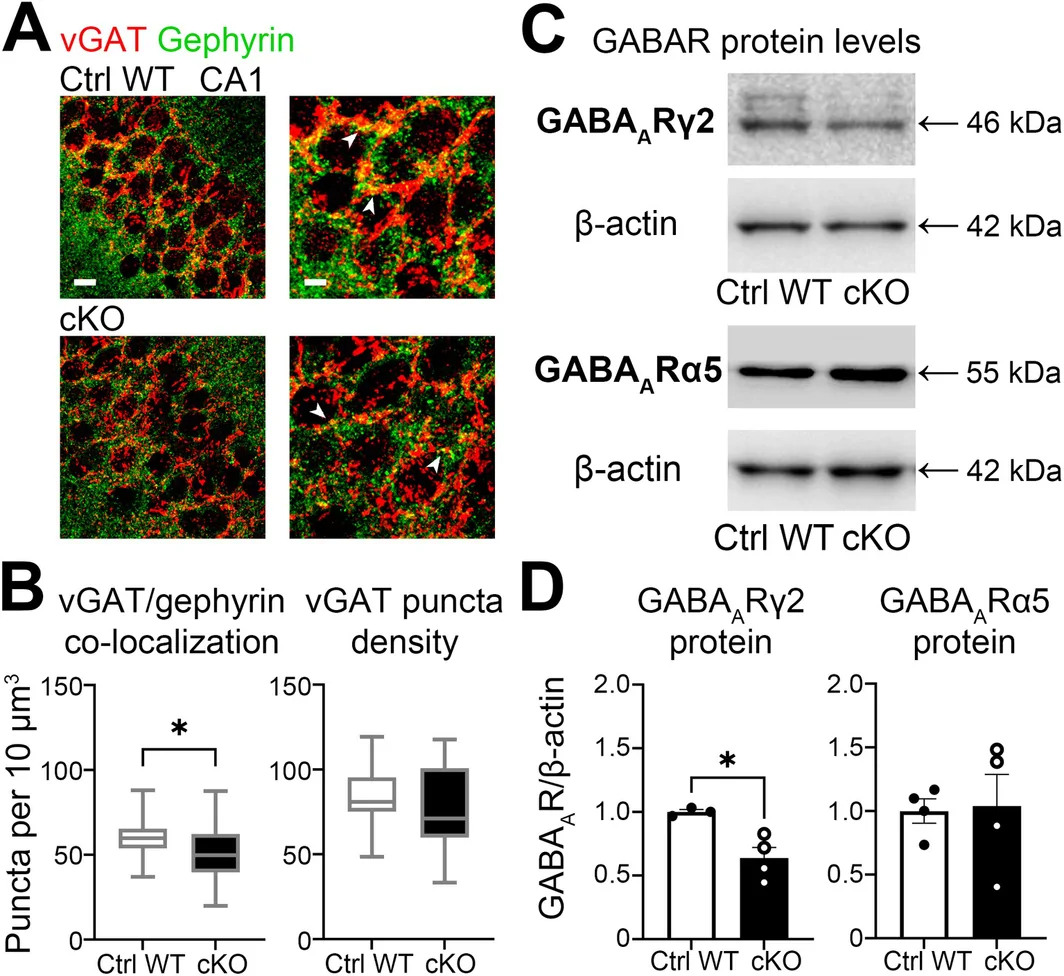
*Fmr1* deletion only from astrocytes impairs inhibitory synaptic connectivity in the CA1 hippocampus of cKO mice. (A) Confocal images showing immunolabeled inhibitory synaptic sites in the SP layer of CA1 HP of P28 Ctrl WT (top) and cKO (bottom) mice. Presynaptic boutons were visualized with vGAT (red) and postsynaptic sites with Gephyrin (green), where overlay appears yellow. Scale bar, 10 μm (left), 5 μm (right). Arrowheads indicate co‐localized puncta. (B) Plots show median and quartiles for density of colocalized vGAT and Gephyrin puncta (left), depicting structural inhibitory synapses, and vGAT puncta (right) (*n* = 31–34 images/4 mice per group, *p* = 0.0297; *t*(63) = 2.224 for vGAT/Gephyrin, *t*(62) = 1.339 for vGAT puncta density; two‐tailed *t*‐test, Table S2). We found a significant reduction in vGAT/Gephyrin co‐localized puncta in CA1 HP of cKO mice compared to Ctrl WT. (C) Western blots showing GABA_A_Rγ2 (~46 kDa), GABA_A_Rα5 (~55 kDa), and beta‐actin (~42 kDa) protein levels in lysates from HP of Ctrl WT and cKO mice. (D) Quantification of GABA_A_R protein levels. One value was excluded based on its identification as an outlier by Grubbs' test (*α* = 0.05). Graphs show mean ± SEM (*n* = 3–4 mice/group, *p* = 0.0158; *t*(5) = 3.585 for GABA_A_Rγ2, *p* = 0.8874; *t*(6) = 0.1477 for GABA_A_Rα5; two‐tailed *t*‐test, Table S2). While we observed a reduction in GABA_A_Rγ2 protein levels, no differences were observed in GABA_A_Rα5 protein levels in HP of cKO mice compared to Ctrl WT.

Reduced expression of synaptic GABA_A_R subunits might reflect fewer inhibitory synapses or fewer GABA receptors per synapse. Therefore, we next investigated whether FMRP deletion from astrocytes during the postnatal period of inhibitory circuit refinement affects inhibitory connectivity. We detected inhibitory synapses by immunolabeling pre‐synaptic vGAT and postsynaptic gephyrin puncta in CA1 HP and analyzing their colocalization (Figure 2A). We observed a significant decrease in the density of colocalized VGAT/Gephyrin puncta in the stratum pyramidal (SP) of CA1 HP of cKO mice compared to Ctrl WT, suggesting reduced perisomatic inhibitory innervation of pyramidal cells (PC) (Figure 2B, Table S2).

To observe how reduced inhibitory synapse connectivity affected GABA_A_R, we next measured protein levels of selected synaptic and extra‐synaptic GABA_A_R subunits, GABA_A_Rγ2 and GABA_A_Rα5, respectively (Figure 2C). The western blot experiments were repeated with an additional group of mice with similar results. Consistent with the reduced mRNA levels, we also observed a significant decrease in the protein levels of the synaptic GABA_A_Rγ2 subunit but not the extrasynaptic GABA_A_Rα5 subunit in cKO mice compared to Ctrl WT (Figure 2D, Table S2). Both reduced inhibitory synaptic sites and synaptic GABA_A_Rγ2 subunit levels suggest impaired inhibitory drive onto PCs.

### 3.3 Amplitude of sIPSCs Is Increased in CA1 Pyramidal Cells Following Astrocyte‐Specific *Fmr1* Deletion

**FIGURE 3:**
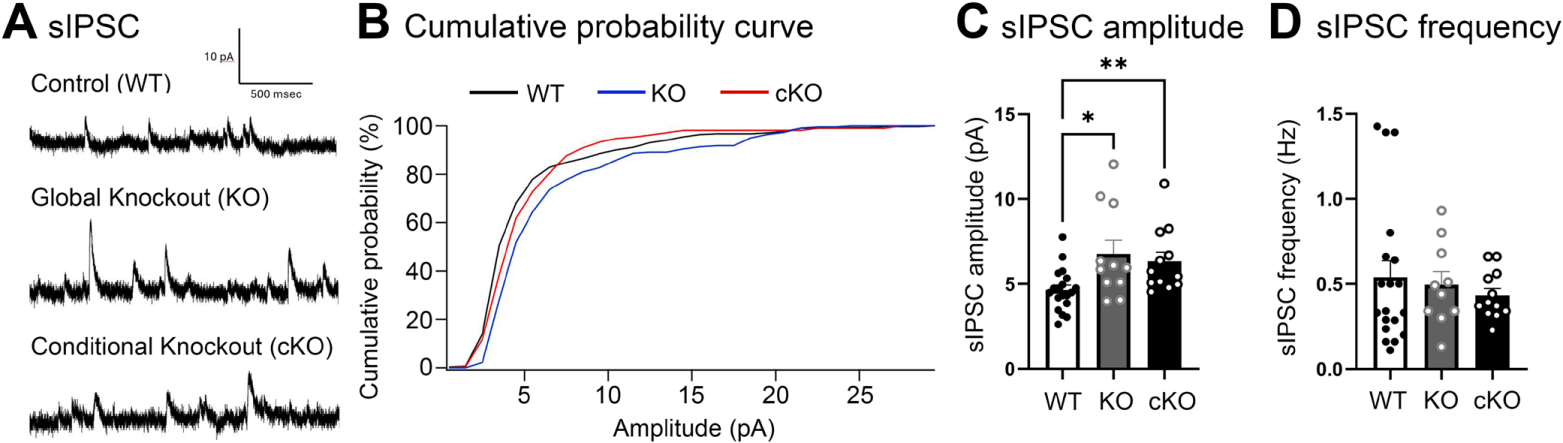
Amplitude but not frequency of spontaneous IPSCs is elevated in CA1 hippocampal neurons of global and astrocyte‐specific *Fmr1* KO mice. (A) Representative traces showing sIPSCs obtained with whole‐cell recordings performed by blind cell patching CA1 pyramidal cells in the hippocampal slices of control (WT), global *Fmr1* KO (KO), and conditional KO (cKO) mice. (B) The cumulative distribution of sIPSC amplitude in WT, KO, and cKO mice. (C) Average sIPSC amplitude is significantly increased in KO and cKO mice compared to WT. Graph shows mean ± SEM (WT *n* = 19 cells/10 mice, KO *n* = 11 cells/5 mice, cKO *n* = 12 cells/8 mice; *p < 0.05; **p < 0.01; Kruskal–Wallis Test, Dunn's post hoc test, *p* = 0.0037; Table S3). (D) Graph shows mean sIPSC frequencies. sIPSC frequency is not significantly different between WT, KO, and cKO groups. One value was excluded based on its identification as an outlier by Grubbs' test (Alpha = 0.05). Graph shows mean ± SEM (WT *n* = 19 cells/10 mice, KO *n* = 11 cells/5 mice, cKO *n* = 12 cells/8 mice; *p < 0.05; **p < 0.01; Kruskal–Wallis Test, *p* = 0.8189; Table S3).

Since we observed reduced overall inhibitory input onto PCs in our cKO model, we next investigated how inhibitory currents onto PCs would be affected. We recorded spontaneous inhibitory postsynaptic currents (sIPSCs) by whole cell recording of PCs from CA1 SP layer in control (WT), global KO (KO), and cKO mice (Figure 3A). Surprisingly, we observed significantly increased sIPSC amplitude in both global KO and cKO mice compared to WT (Figure 3B,C, Table S3), and no significant changes in sIPSC frequency (Figure 3D, Table S3). For these experiments, we used both tamoxifen administered Ctrl WT and un‐injected WT and found no significant differences between the un‐injected WT and Ctrl WT groups (Table S3). Since we did not see any difference between the groups, the data were combined for greater power during statistical analysis. While we see a right shift in cumulative curve in KO, suggesting increased proportion of high amplitude events, this was less evident in cKO mice. The increase in the sIPSC amplitude observed in cKO may be a result of excess buildup of extracellular GABA following its synaptic release, which would result in prolonged activation of GABA receptors enhancing sIPSC amplitude (Edwards et al. 1990; Field et al. 2021).

### 3.4 Tonic GABA Currents Are Reduced in CA1 Pyramidal Cells of Global *Fmr1* KO but Not cKO

**FIGURE 4:**
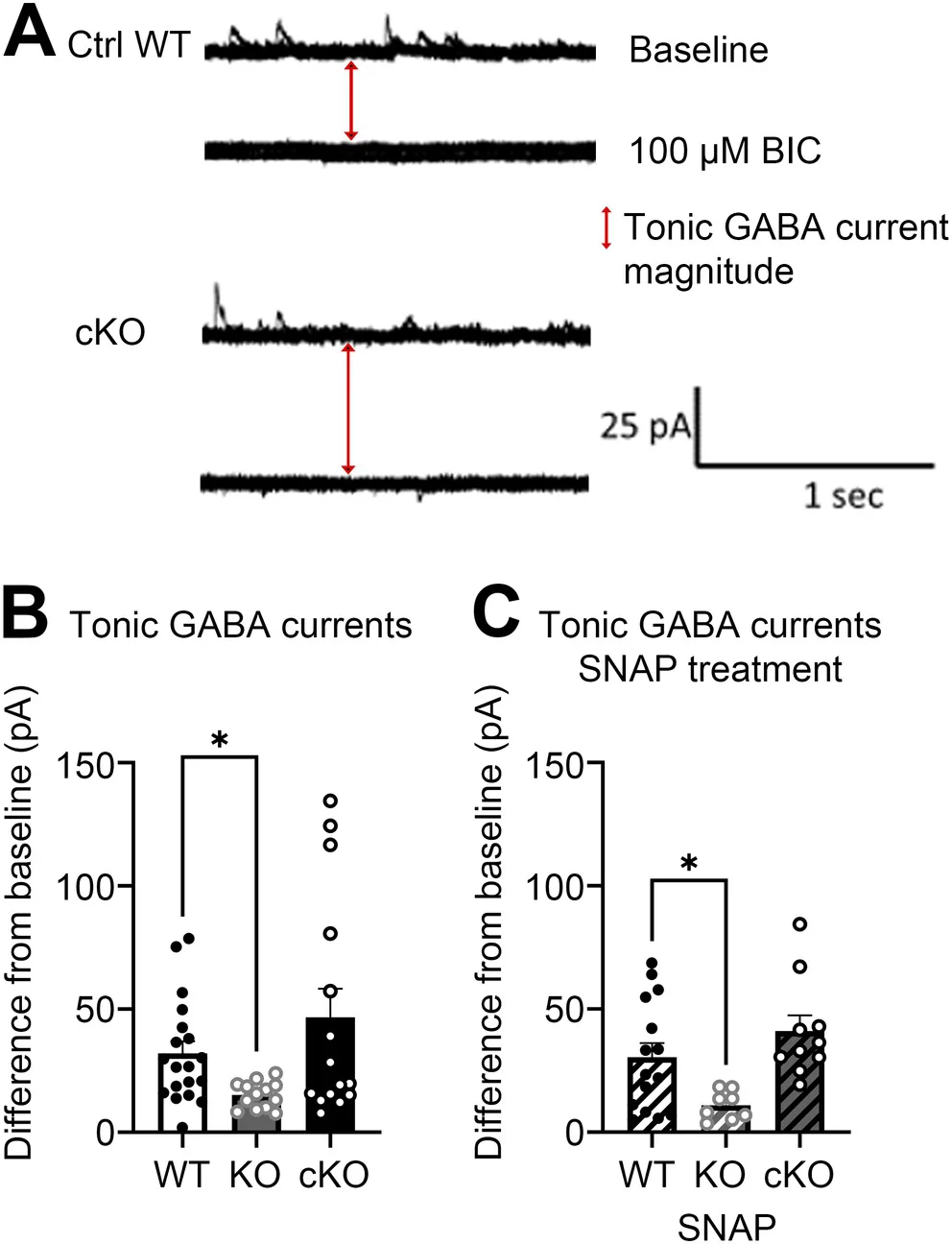
Tonic currents were reduced in global KO but not cKO mice. (A) Representative traces showing baseline currents before and after bicuculline (BIC), where the difference in baseline current represents the magnitude of tonic GABA current, obtained with whole‐cell recordings performed by blind cell patching pyramidal cells in the CA1 HP. (B) Tonic GABA currents in CA1 pyramidal cells in hippocampal slices of WT, global Fmr1 KO and conditional KO (cKO) mice. Graphs show mean ± SEM (WT *n* = 19 cells/10 mice, KO *n* = 13/5 mice, cKO *n* = 15 cells/8 mice; *p < 0.05; one‐way ANOVA, Kruskal–Wallis's post hoc test; Genotype: *p* = 0.0206; Table S4). Tonic GABA currents are reduced in global Fmr1 KO compared to Ctrl WT and cKO. Tonic GABA currents are not significantly different in cKO compared to Ctrl WT. (C) Tonic GABA currents in the presence of SNAP in CA1 pyramidal cells in hippocampal slices of WT, global Fmr1 KO and conditional KO (cKO) mice. Graphs show mean ± SEM (WT SNAP *n* = 15 cells/10 mice, global KO SNAP *n* = 8/4 mice, cKO SNAP *n* = 10 cells/6 mice; *p < 0.05; Brown‐Frosythe ANOVA test, Dunn's post hoc test; Genotype: *p* = 0.0039; Table S4). Tonic GABA currents are reduced in SNAP‐treated global Fmr1 KO compared to SNAP‐treated WT. Tonic GABA currents are not significantly different in astrocyte cKO compared to WT after SNAP treatment.

Tonic inhibition is proposed to gate spatial memory, sensory processing, and motor coordination (Shen et al. 2024; Tang et al. 2011; Woo et al. 2018) and its dysregulation has been identified in Rett Syndrome, epilepsy, Parkinson's, and Alzheimer's (Chazalon et al. 2018; Cope et al. 2009; Dong et al. 2020; Wu et al. 2014; Zhang et al. 2007). Previous studies have shown reduced tonic inhibition in hippocampal excitatory neurons in global *Fmr1* KO, attributed to reduced expression of extrasynaptic GABA _A_Rs (Curia et al. 2009; Modgil et al. 2019). As we observed changes in phasic inhibition in astrocyte‐specific cKO, we next investigated how tonic inhibition of PCs was impacted in cKO compared to global KO mice (Figure 4A). Similar to prior studies, we also observed significantly reduced tonic GABA currents in CA1 PCs of global KO compared to WT mice (Figure 4B, Table S4), most likely due to the loss of extrasynaptic GABA _A_Rs. However, no significant differences were observed in tonic GABA currents on CA1 PCs of cKO that also showed normal levels of extrasynaptic GABA_A_Rα5 subunit, with a potential trend in the opposite direction (Figure 4B, Table S4). For these experiments, we also used both tamoxifen administered Ctrl WT and un‐injected WT and found no significant differences between the un‐injected WT and Ctrl WT groups (Table S3). Since we did not see any difference between the groups, the data were combined for greater power during statistical analysis.

GABA transport in astrocytes can modulate tonic inhibition (Kersanté et al. 2013; Kwak et al. 2020; Yang et al. 2023). Therefore, we next investigated the role of astrocytic GABA transport in tonic inhibition in our cKO mouse model by pharmacologically blocking astrocyte‐specific GABA transporter GAT3. We observed no changes in tonic inhibition of CA1 PCs in the hippocampal slices of global KO and cKO mice in the presence of GAT3 antagonist SNAP‐5114 (SNAP) compared to the WT group (Figure 4C, Table S4). Our findings indicate that tonic inhibition is reduced in CA1 PCs in the hippocampus of global *Fmr1* KO mice, but not in cKO mice, likely explained by differences in extrasynaptic GABA _A_R expression, and that blocking astrocyte GABA transport does not affect tonic inhibition on PCs in the CA1 hippocampus of global KO and cKO mice.

### 3.5 Astrocyte‐Specific *Fmr1* Deletion Affects GABA Transport in Astrocytes

**FIGURE 5:**
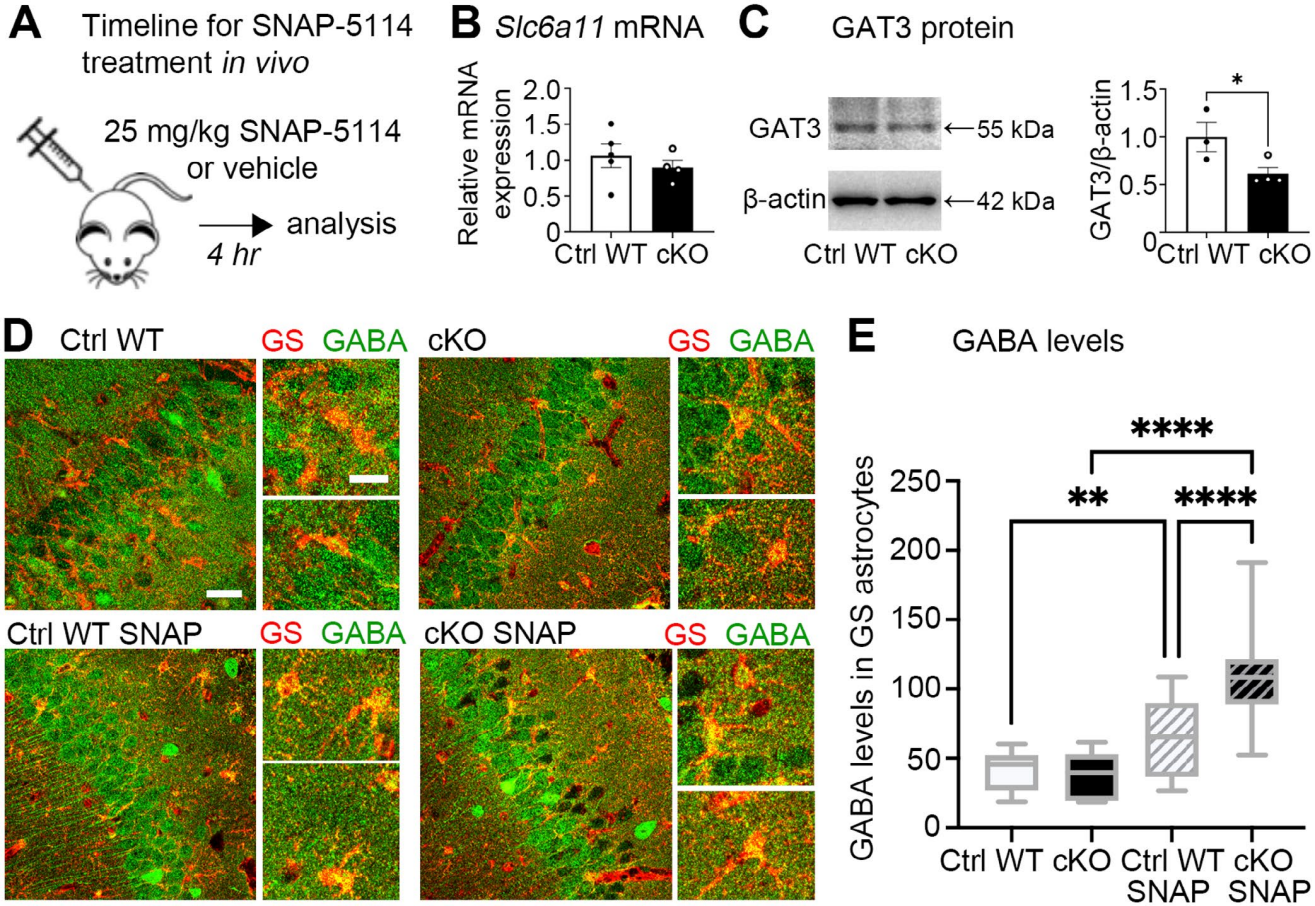
CA1 hippocampal astrocytes exhibit elevated levels of GABA following the inhibition of GABA transport in astrocytes with SNAP. (A) Graphics showing experimental timeline for acute treatment with either GAT3 inhibitor SNAP‐5114 or vehicle in vivo. (B) Quantitative analysis of GAT3‐encoding gene *Slc6a11* mRNA performed with qRT‐PCR. Graph shows mean ± SEM (*n* = 4–5 mice/group; *p* = 0.05; *t*(7) = 0.7896, two‐tailed *t*‐test, Table S5). We found no significant differences in *Slc6a11* expression in cKO mice compared to Ctrl WT. (C) Western blots showing GAT3 (~55 kDa) and beta‐actin (~42 kDa) protein levels in HP lysates from Ctrl WT and cKO mice. Graph shows mean ± SEM (*n* = 3–4 mice/group; *p < 0.05; *t*(5) = 2.576, two‐tailed *t*‐test, Table S5). There is a significant decrease in astrocytic GABA transporter GAT3 protein levels in cKO mice compared to Ctrl WT. (D) Confocal images showing Glutamine Synthetase (GS, red) and GABA (green) immunoreactivity in CA1 HP of vehicle‐treated and SNAP‐treated Ctrl WT and cKO mice. Scale bar, 30 μm in full size image and 15 μm in high magnification image. (E) Quantitative analysis of the intensity of GABA immunoreactivity in GS‐labeled astrocytes in CA1 HP. Graphs show median with quartiles (*n* = 18 images/3 mice per group, **p < 0.01, ****p < 0.0001; two‐way ANOVA, Uncorrected Fisher's LSD post hoc test, Genotype: *F*(1, 68) = 10.81, *p* = 0.0016; Treatment: *F*(1, 68) = 70.31, *p* < 0.0001; Interaction: *F*(1, 68) = 16.00, *p* = 0.0002; Table S5). GABA levels in GS‐labeled astrocytes were significantly increased in SNAP groups compared to vehicle controls and in cKO SNAP mice compared to Ctrl WT SNAP mice.

In the hippocampus, astrocytes usually uptake GABA, and inhibition of GABA transport in astrocytes can lead to increased levels of extracellular GABA (Kersanté et al. 2013; Yang et al. 2023). We did not see an effect of blocking astrocyte GABA transport on tonic inhibition of CA1 PCs in the hippocampal slices of cKO mice. However, reduced GABA uptake by astrocytes can contribute to excess extracellular GABA, causing a prolonged GABA receptor activation, associated with increased amplitude of sIPSCs in PCs of cKO. Therefore, we next assessed GABA transport in astrocytes by analyzing the expression of astrocytic GABA transporter GAT3 in the hippocampus and the effects of its inhibition in vivo with SNAP 5114 (Figure 5A). Although we found no differences in mRNA expression of GAT3‐encoding gene *Slc6a11* with qRT‐PCR (Figure 5B, Table S5), there was a significant reduction in GAT3 protein levels in HP lysates (Figure 5C, Table S5). We have previously performed high‐performance liquid chromatography (HPLC) using FXS patient‐derived induced pluripotent stem cells differentiated into astrocytes in vitro, which showed increased GABA levels in cultured FXS astrocytes (Wagner et al. 2026). Performing HPLC in mouse brain tissue would mask the astrocyte‐dependent changes in extracellular GABA since the majority of the GABA comes from neurons, making the interpretation of the results difficult. Therefore, intracellular GABA immunolabeling was performed using an approach previously utilized by several investigators (Yoon et al. 2011; Ormel et al. 2020). The staining protocol was identical among groups, processing was done in mixed groups, and analysis was done separately by a different experimenter to minimize any bias. If astrocytes primarily uptake GABA, then the inhibition of GABA transport should result in either a decrease or no change in GABA levels in cKO astrocytes, which show reduced GAT3 levels. However, we found significantly elevated GABA levels in hippocampal astrocytes of SNAP‐treated cKO compared to vehicle‐treated cKO groups. Although we also observed slightly increased astrocyte GABA levels in SNAP‐treated WT, GABA levels were significantly higher in SNAP‐treated cKO compared to the SNAP‐treated WT group (Figure 5D,E, Table S5). Further analysis of GABA levels in astrocytes revealed that only 20% of astrocytes showed elevated GABA in the SNAP‐treated WT group, while the majority of astrocytes (70%) in SNAP‐treated cKO exhibited a significant increase in GABA levels. Overall, these findings suggest an abnormal GABA transport in cKO astrocytes, potentially due to increased GABA synthesis (Wagner et al. 2026), leading to excess of extracellular GABA.

### 3.6 Abnormal GABA Transport in Astrocytes Suppresses PV Expression in cKO Mice

**FIGURE 6:**
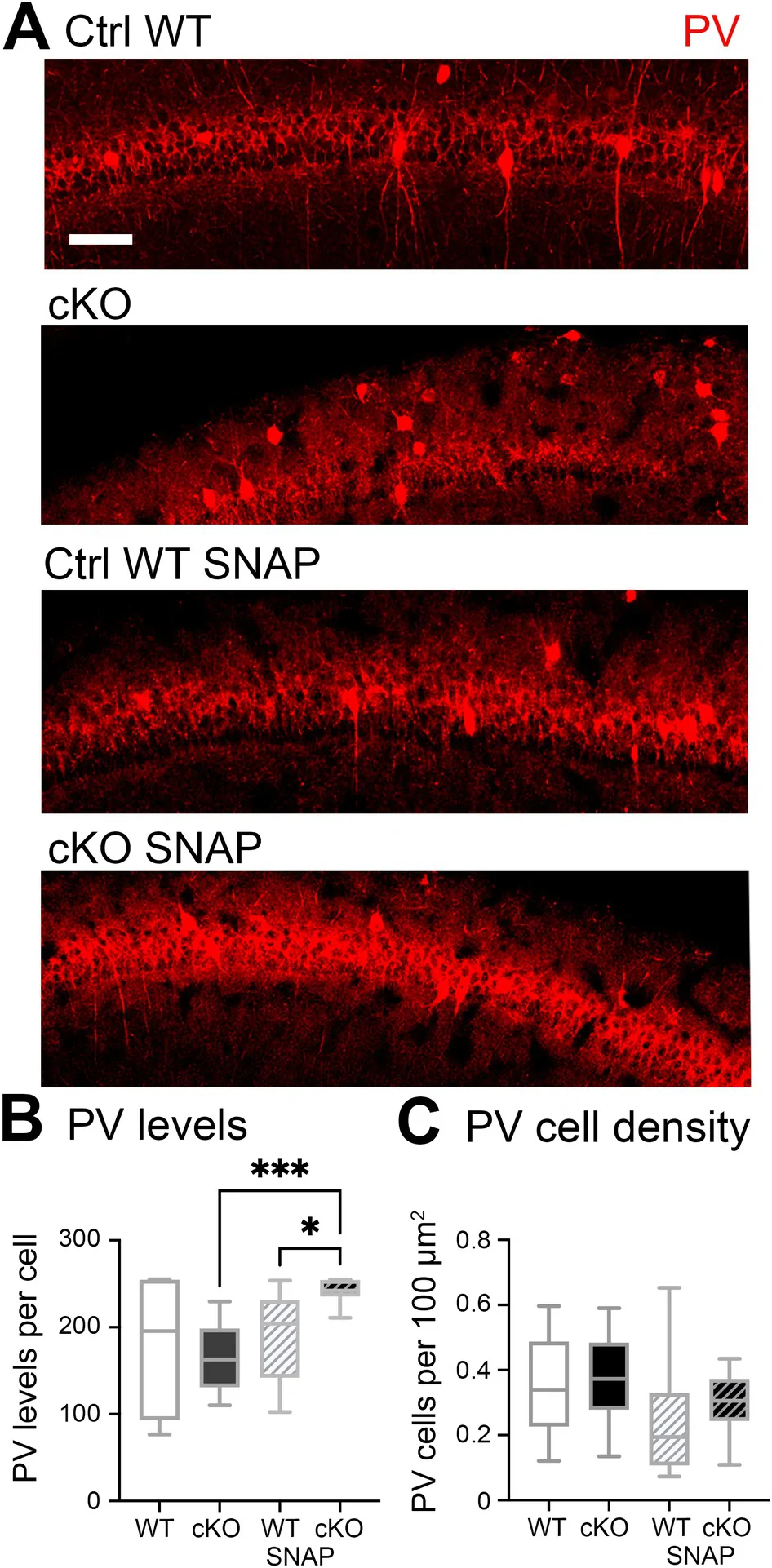
Inhibition of GABA transport in astrocytes enhances PV expression in cKO but not WT mice. (A) Confocal images showing immunolabeled PV cells in CA1 HP of P28 Ctrl WT and cKO mice with either vehicle or SNAP treatment. Scale bar, 70 μm. (B) PV levels are assessed by measuring mean intensity of PV immunoreactivity per cell. Plot shows median and quartiles (*n* = 12–16 images/3 mice per group, *p < 0.05; two‐way ANOVA, Uncorrected Fisher's LSD post hoc test, Genotype: *F*(1, 48) = 1.533, *p* = 0.2216; Treatment: *F*(1, 48) = 9.247, *p* = 0.0038; Interaction: *F*(1, 48) = 4.596, *p* = 0.0371; Table S6). PV levels are significantly decreased in cKO mice after SNAP treatment. (C) PV cell density per 100 μm^2^. Plot shows median and quartiles (*n* = 12–20 images/3 mice per group, *p* = 0.05; two‐way ANOVA, Tukey's post hoc test, Genotype: *F*(1, 60) = 0.9561, *p* = 0.3321; Treatment: *F*(1, 60) = 5.210, *p* = 0.0260; Interaction: *F*(1, 60) = 0.1383, *p* = 0.7113; Table S6). No significant differences were found between groups in PV cell density.

While we did not see effects of altered GABA transport in cKO astrocytes on tonic inhibition of PCs in hippocampal slices, PV cells may be more sensitive to tonic inhibition (Kinney 2005; Bryson et al. 2020). As PV protein is expressed in an activity dependent manner (Kamphuis et al. 1989; Patz et al. 2004; Donato et al. 2013), we next investigated PV levels as a metric for PV activity. Although we observed no changes in PV levels or PV cell density in CA1 HP of cKO mice compared to Ctrl WT (Figure 6A–C, Table S6), in the presence of GAT3 antagonist SNAP, we found significantly higher PV levels in SNAP‐treated cKO hippocampus compared to SNAP‐treated WT group (Figure 6A,B, Table S6). PV levels in SNAP‐treated cKO were also significantly higher than in vehicle‐treated cKO group, whereas the treatment did not affect PV levels in WT group (Figure 6A,B, Table S6). These findings suggest that elevated GABA levels in cKO hippocampal astrocytes not only affect extracellular GABA uptake, enhancing phasic inhibition on PCs, but at the same time may regulate PV cell activity in CA1 HP. Our findings suggest the dysregulation of PV cell activity in the hippocampus of cKO mice via abnormal GABA transport in astrocytes.

### 3.7 Postnatal Deletion of *Fmr1* in Astrocytes Leads to Reduced Socialization and Impaired Spatial Memory in cKO Mice

**FIGURE 7:**
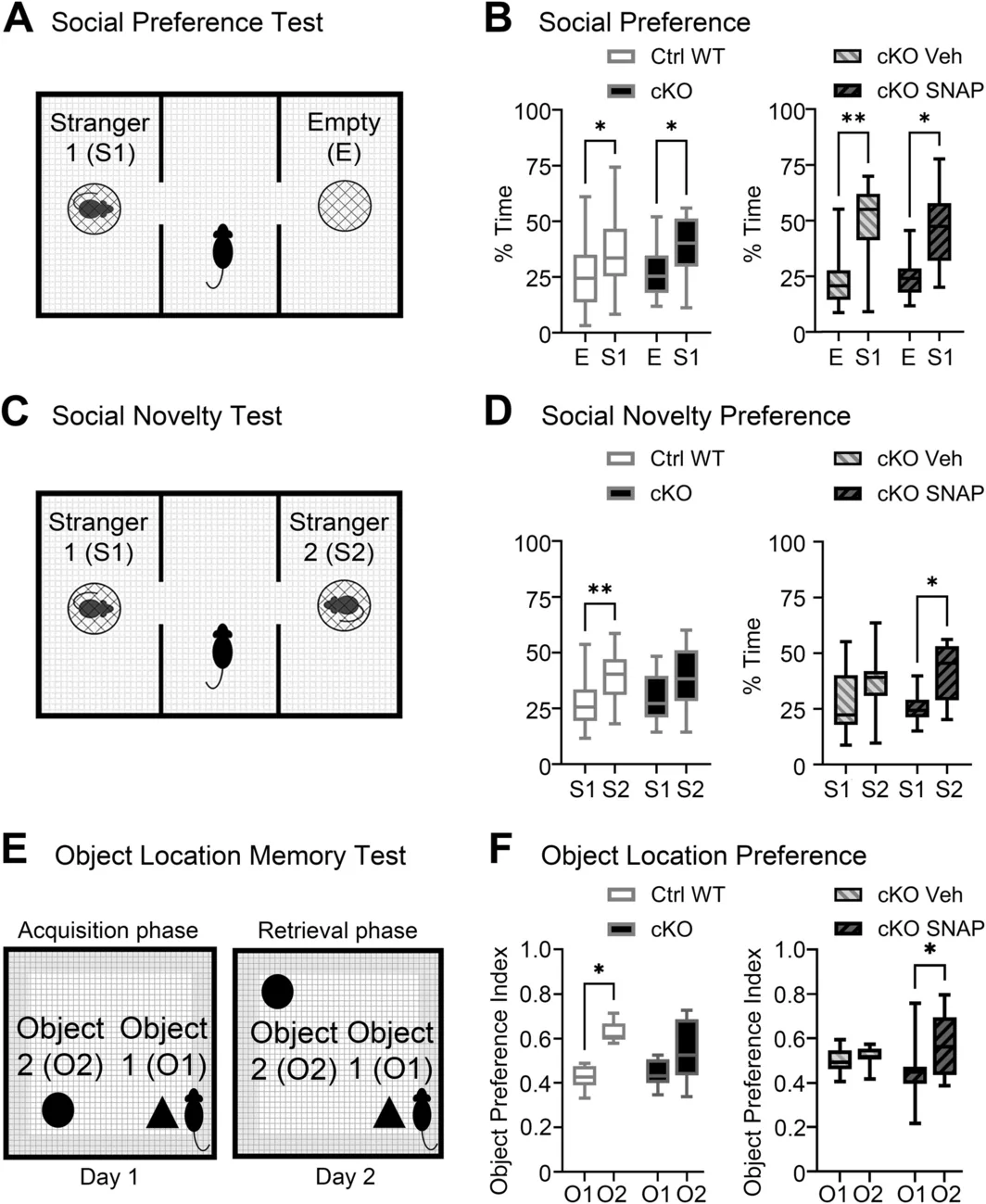
Impaired object location memory and social novelty preference were rescued in cKO mice following acute inhibition of GAT3‐mediated GABA transport in astrocytes. (A) Mice are first exposed to a 3‐chamber assay with one side chamber containing a stranger mouse (S1) and the second side chamber remains empty (E). (B) Graphs demonstrate the performance of Ctrl WT (*n* = 29), cKO (*n* = 20), and cKO SNAP (SNAP, *n* = 10), and cKO vehicle (Veh; *n* = 13) mice in the social preference test. Social preference in the first 5 min of the test is indicated by percentage of time spent with S1 mouse compared to the empty chamber. WT and cKO mice spend significantly more time with S1 than the empty chamber. Graph shows median and quartiles (**p < 0.01, ****p < 0.0001; two‐way ANOVA, Bonferroni's multiple comparisons test, Stranger: *F*(1, 47) = 13.27, *p* = 0.0007; Genotype: *F*(1, 47) = 1.095, *p* = 0.3007; Interaction: *F*(1, 47) = 0.1731, *p* = 0.1731; Table S7). Both cKO, vehicle‐treated and SNAP treated mice also spent significantly more time with S1 than the empty chamber. Graph shows median and quartiles (*p < 0.05; two‐way ANOVA, Bonferroni's multiple comparisons test, Stranger: *F*(1, 21) = 18.00, *p* = 0.0004; Treatment: *F*(1, 21) = 0.1474, *p* = 0.7049; Interaction: *F*(1, 21) = 0.2143, *p* = 0.6482; Table S7). (C) In the second phase of the test, a novel mouse (S2) is added to the second side chamber, while S1 remains in the first side chamber. (D) Graphs demonstrate the performance of Ctrl WT (*n* = 29), cKO (*n* = 23), cKO SNAP (SNAP, *n* = 10), and cKO veh (Veh, *n* = 14) mice in the social novelty preference test. Social novelty preference in the first 5 min of the test is indicated by percentage of time spent with novel S2 compared to familiar S1 mouse. Ctrl WT but not cKO mice spent significantly more time with S2 than S1. Graph shows median and quartiles (*p < 0.05, **p < 0.01; two‐way ANOVA, Bonferroni's multiple comparisons test, Stranger: *F*(1, 49) = 11.86, *p* = 0.0012; Genotype: *F*(1, 49) = 0.2565, *p* = 0.6148; Interaction: *F*(1, 49) = 0.4854, *p* = 0.4893; Table S7). SNAP treatment improved social novelty preference as cKO SNAP but not cKO veh mice show a significant preference in time spent with S2. Graph shows median and quartiles (*p < 0.05; two‐way ANOVA, Bonferroni's multiple comparisons test, Stranger: *F*(1, 22) = 10.16, *p* = 0.0043; Treatment: *F*(1, 22) = 0.2608, *p* = 0.6147; Interaction: *F*(1, 22) = 0.5767, *p* = 0.4557; Table S7). (E) Mice are first exposed on day 1 to two objects (O1 & O2) with different size and texture placed on two corners of the same half of the open arena. The location of object 2 (O2) is moved on Day 2 to the corner on the other half of the open arena. Mice explore the objects for 10 min each day. Preference index > 0.5 indicates preference for the object on day 2 compared to Day 1, while index less or equal to 0.5 indicates no preference. (F) Graph shows median and quartiles (*p < 0.05; Tukey's multiple comparisons post hoc test, Object: *F*(1, 8) = 29.94, *p* = 0.0006; Genotype: *F*(1,8) = 1.225, *p* = 3.006; Interaction: *F*(1,3) = 2.997, *p* = 0.1819; Table S7). Ctrl WT, but not cKO show a higher preference for O2 than O1 on day 2. Graph shows median and quartiles (*p < 0.05; Uncorrected Fisher's LSD post hoc test, Object: *F*(1, 38) = 0.008622, *p* = 0.9265; Treatment: F(1,38) = 1.796, *p* = 0.1882; Interaction: *F*(1,38) = 3.693, *p* = 0.0621; Table S7). SNAP‐treated cKO mice show a significantly higher preference for O2 compared to O1 on Day 2, where vehicle‐treated cKO show no preference for O1 or O2.

Altered inhibition in the hippocampus may impact hippocampal‐dependent behaviors, such as social novelty preference and spatial memory, and account for social and spatial learning deficits observed in individuals with FXS (Hitti and Siegelbaum 2014). Particularly, PV cells have been implicated in spatial memory and reversal learning (Ognjanovski et al. 2017; Jetsonen et al. 2023). Therefore, we next assessed sociability, social novelty preference, and spatial memory using a three‐chamber test and object location memory test respectively (Figure 7). We found intact social preference with no genotype differences in sociability (Figure 7A,B, Table S7) but impaired social novelty preference in cKO mice (Figure 7C,D, Table S7). Ctrl WT spent significantly more time with a novel stranger compared to a familiar mouse, while cKO mice spent comparable time with both mice (Figure 7D, Table S7). Vehicle‐treated cKO mice also failed to show a preference for a novel mouse, which was rescued in SNAP‐treated cKO mice, similar to the significant preference observed in Ctrl WT (Figure 7D, Table S7).

The beneficial effects of SNAP treatment were also observed in spatial memory of cKO in the object location memory test. During the spatial memory retrieval phase on Day 2, Ctrl WT mice showed a higher preference for the moved object 2 (O2) compared to the unmoved object 1 (O1), while cKO did not show a significant preference (Figure 7F, Table S7). This highlights intact spatial memory in Ctrl WT, and not in cKO. Similarly, vehicle‐treated cKO mice also did not show a preference for O2 on Day 2. However, SNAP‐treated cKO spent more time exploring O2, similar to the Ctrl WT mice (Figure 7F, Table S7).

Our findings indicate that abnormal GABA transport in *Fmr1* KO astrocytes contributes to sociability and spatial memory deficits observed in the mouse model of FXS.

## 4 Discussion

**FIGURE 8:**
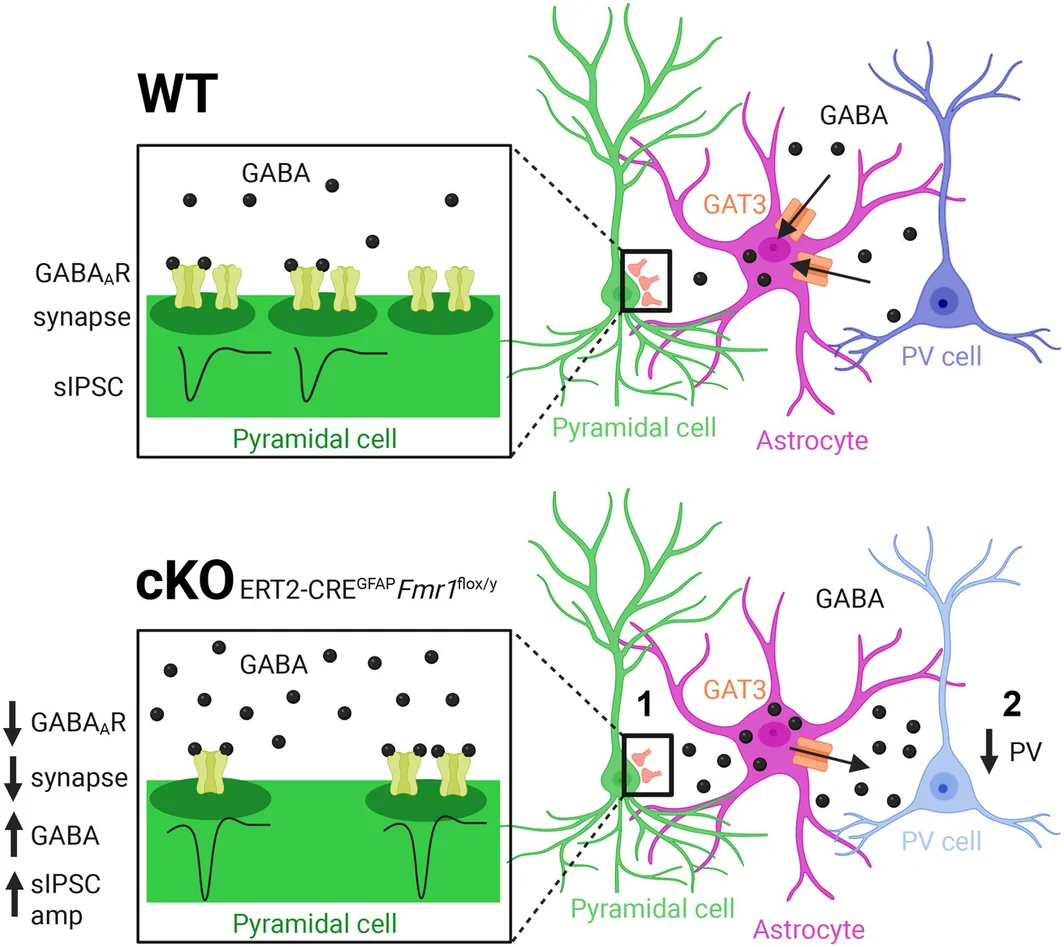
Schematic depiction of phenotypes observed in the hippocampus following deletion of *Fmr1* from astrocytes during P14–P28 postnatal developmental period compared to Ctrl WT. Deletion of *Fmr1* from astrocytes (cKO) during P14–P28 postnatal developmental period affects (1) perisynaptic GABAergic synapses on CA1 pyramidal cells (box) and (2) PV levels (light blue) most likely due to excess GABA (black dots) in cKO astrocytes (Wagner et al. 2026), leading to impaired GABA uptake by astrocytes (arrow in) and potentially reverse GABA transport from astrocytes (arrow out) as blocking astrocytic GABA transporter GAT3 (orange) results in accumulation of GABA in cKO astrocytes. Insert depicts changes at GABAergic synapses on pyramidal neuron, including reduced synaptic GABA_A_R (yellow), decreased number of perisynaptic GABAergic post‐synaptic sites (dark green circles), but increased sIPSC amplitude (black lines). The drawing was created in BioRender.

The results of this study suggest a non‐neuronal mechanism of abnormal inhibitory hippocampal circuit development in FXS. We have shown that deletion of *Fmr1* from astrocytes during the postnatal developmental period of inhibitory circuit refinements affects (1) development of GABAergic synapses; (2) phasic but not tonic inhibitory currents in pyramidal neurons; (3) PV levels; and (4) sociability and spatial memory; potentially through impaired GABA transport in astrocytes (Figure 8).

FMRP is shown to target the mRNAs encoding eight different GABA_A_R subunits (α1, α3, α4, β1, β2, γ1, γ2, and δ) (D'Hulst et al. 2006; Olmos‐Serrano et al. 2010; Sabanov et al. 2017; Zhang et al. 2007), which are reduced in global *Fmr1* KO hippocampus (Deng et al. 2022; El Idrissi et al. 2005; Sabanov et al. 2017) and in humans with FXS (Braat et al. 2015). Our findings demonstrate that astrocyte‐specific deletion of *Fmr1* also leads to reduced expression of synaptic GABA_A_R subunits α1, α3, and γ2, indicating that the reduced GABA_A_R levels cannot be explained by direct interaction between FMRP and GABA_A_R mRNA in neurons. Potentially, reduced GABA_A_R levels simply reflect the overall reduction in inhibitory synaptic sites we observed in our model. Astrocytes have established roles in synapse formation and elimination (Chung et al. 2015; Irala et al. 2024; Park and Chung 2023) and could contribute to inhibitory synapse impairments in FXS through several different mechanisms. Firstly, synapse formation relies heavily on astrocyte‐dependent regulation of cholesterol (van Deijk et al. 2017; Zhang et al. 2015), which has been shown to be impaired in FXS (Talvio et al. 2023). Secondly, microglia can use GABA as a cue for pruning inhibitory synapses (Favuzzi et al. 2021) and potentially could be recruited by the excess extracellular GABA.

We also observed increased sIPSC amplitude, but not frequency, in CA1 PC in both global KO and our cKO model, suggesting this feature is due to an astrocytic mechanism. It is possible that elevated extracellular GABA due to impaired uptake of GABA by KO astrocytes may contribute to prolonged activation of GABA receptors, resulting in increased amplitude. Increased sIPSC amplitude may also arise from altered postsynaptic GABA receptor sensitivity, changes in postsynaptic scaffold, such as altered gephyrin‐dependent stabilization of synaptic GABA_A_ receptor clusters, or altered presynaptic GABA release probability (Banks et al. 2002; Nusser et al. 1997; Tyagarajan and Fritschy 2014; Luscher et al. 2011). Previous work has also shown increased amplitude but not frequency of sIPSCs in dentate gyrus granule cells (DGGCs) of P21–P35 *Fmr1* KO mice, similar to our findings (Modgil et al. 2019; Deng et al. 2024). A plethora of others investigating phasic inhibition onto excitatory neurons in FXS have found that changes in amplitude and frequency of sIPSCs vary by age and brain region (Cea‐Del Rio et al. 2020; Centonze et al. 2008; Curia et al. 2009; Deng and Klyachko 2016; Kang et al. 2017; Olmos‐Serrano et al. 2010; Paluszkiewicz et al. 2011; Sabanov et al. 2017; Svalina et al. 2022).

sIPSC frequency is informed both by density of inhibitory synapses as well as firing rate of presynaptic interneurons (Hijazi et al. 2020; Kobayashi and Buckmaster 2003; Shao et al. 2012; Trotter et al. 2006) and was unchanged in our model despite reduced inhibitory synaptic sites observed in cKO mouse. Previous work from our lab indicates FXS cortical astrocytes have elevated GABA due to increased GABA synthesizing enzyme GAD65/67 (Wagner et al. 2026), which can be attributed to FMRP indirectly regulating translation of *gad2 gene* through a miR‐31‐5p mechanism (Men et al. 2020). Astrocytes regulate extracellular GABA levels by up‐taking GABA through GAT3 (Kersanté et al. 2013); however, elevated astrocytic GABA can shift the concentration gradient to prevent GABA uptake through GAT3, and even result in spillover of GABA into the extracellular space (Dvorzhak et al. 2010; Héja et al. 2009; Kilb and Kirischuk 2022; Kinney 2005). Impaired GABA uptake by astrocytes, either by inhibiting GAT3 or by elevating astrocytic GABA, weakens contextual fear memory and spatial memory, respectively (Park et al. 2025; Shen et al. 2024). GAT3‐mediated GABA uptake results in calcium waves in astrocytes that trigger release of neurotransmitters that enhance synaptic transmission through the activation of NMDA receptors, and this mechanism of GABA reception is critical for dorsal hippocampal functions such as learning and memory (Boddum et al. 2016; Shen et al. 2024). Targeting excessive GABA synthesis in astrocytes may be more effective in rescuing the synaptic and behavioral impairments we observed.

Our observations of tonic inhibition in global KO mice are consistent with literature showing tonic GABA is reduced in excitatory neurons in the hippocampus in FXS (Curia et al. 2009; Modgil et al. 2019; Sabanov et al. 2017), most likely due to reduced levels of extrasynaptic GABA_A_Rs. In contrast, cKO mice did not show changes in the levels of extrasynaptic GABA_A_Rs nor tonic inhibition on PCs, which was also not affected by blocking GABA transport in astrocytes. An astrocyte‐specific model of FXS was also shown to have reduced glutamate uptake in the cortex, leading to enhanced neuronal excitability (Higashimori et al. 2016); potentially impaired GABA and glutamate uptake by astrocytes are affecting both inhibitory and excitatory tone. Indeed, previous work in *Fmr1* KO mice found that elevated E/I ratio yielded stable synaptic spiking in the cortex, suggesting robust homeostatic stabilization of network activity (Antoine et al. 2019).

Excess GABA synthesis by *Fmr1* astrocytes can result in impaired uptake of extracellular GABA and prolonged activation of GABA_A_R, explaining elevated sIPSC amplitude. The prolonged activation of GABA_A_R can lead to their desensitization, explaining the overall reduction in GABA_A_R levels and scaling down inhibitory synaptic connections in cKO. As GABA uptake was already impaired in cKO astrocytes, acute inhibition of GABA transport by astrocytes did not significantly affect tonic inhibition of CA1 pyramidal neurons; however, it affected PV expression. Cell type‐specific effects were previously observed with extracellular GABA excess disproportionately inhibiting PV in the somatosensory cortex (Bogaj et al. 2023; Bryson et al. 2020). Our data show that blocking GAT3 resulted in the accumulation of GABA in hippocampal astrocytes and elevated PV expression, suggesting higher PV cell activity. In our model, GABA may suppress PV cell activity potentially through reverse GABA transport in astrocytes (Figure 8), a mechanism that was previously described in rat neocortex, where GAT3 blockade increased inhibitory postsynaptic potentials in cortical neurons (Kinney 2005).

The changes in PV expression following GAT3 blockade in our cKO model most likely contribute to the normalization of spatial memory retrieval in the object location memory test as well as social novelty preference. PV network configuration is shown to critically influence spatial memory consolidation, particularly in spatial memory‐related tasks (Donato et al. 2013; Pérez‐Montoyo et al. 2025). Along with the involvement of PV in memory consolidation and retrieval (Ognjanovski et al. 2017), PV cells are known to engage in plasticity related signaling, such as the TrkB activation, during reversal learning, especially in spatial tasks (Jetsonen et al. 2023). Hence, the ability of PV to mediate both initial spatial memory acquisition, regulate reversal learning and social novelty preference is critical for hippocampal memory flexibility. Olfaction may also affect social behavior and social novelty preference (Thye et al. 2018); and atypical olfactory sensory processing has been observed in individuals with ASDs as well in mouse models of FXS (Tonacci et al. 2017; Rogers et al. 2003). Future studies should be done testing the role of olfaction in impaired social behaviors in FXS mouse models.

## 5 Conclusion

Together, these findings suggest that impaired GABA uptake by *Fmr1* KO astrocytes and potentially reverse GABA transport may both contribute to changes in the development of inhibitory circuits in the hippocampus and underlie impaired spatial memory. In this study we provide indirect evidence of impaired GABA clearance by astrocytes and potential reverse transport of GABA in astrocytes. We are currently implementing a new approach to monitor GABA transients in astrocytes using the iGABASnFR biosensor expressed under the *gfap* promoter. Along with that, in vivo calcium imaging of PV interneurons would be a great addition to measure transient changes in PV activity in vivo in live animals, which we are currently developing in the lab. Overall, future studies will determine whether enhanced PV expression following GAT3 blockade leads to the increase in PV cell activity that is responsible for improved spatial memory retrieval and reversal learning in FXS mouse models.

In summary, we demonstrate an astrocytic mechanism for GABA dysregulation in the hippocampus of an FXS mouse model, with consequences for the structure, function, and behavior, underscoring the role of astrocytic GABA transport in mediating inhibitory circuit development.

## Author Contributions

**Maham Rais:** methodology, validation, formal analysis. **Alexandra Varallo:** methodology, validation, formal analysis. **Sarah Maples:** methodology, formal analysis, writing – review and editing, investigation, validation, software. **Samantha Sutley‐Koury:** methodology, validation, formal analysis. **Jordan Wimberly:** methodology, validation, formal analysis, software, writing – review and editing. **Ritika Thapa:** methodology, validation, formal analysis, writing – review and editing, investigation, software. **Victoria A. Wagner:** conceptualization, investigation, writing – original draft, methodology, validation, writing – review and editing, software, formal analysis. **Peter Hickmott:** methodology, validation, writing – review and editing, formal analysis. **Ellie Pettijohn:** methodology, validation, formal analysis. **Iryna M. Ethell:** conceptualization, funding acquisition, writing – original draft, writing – review and editing, formal analysis, project administration, data curation, supervision, resources.

## Funding

This work was supported by: National Institute of Neurological Disorders and Stroke (NS129555 to I.M.E.). TRANSCEND fellowship from the California Institute of Regenerative Medicine (Award # EDUC4‐12752 to V.A.W.). National Institute of Neurological Disorders and Stroke National Research Service Award Fellowship (F31NS117178 to M.R). National Institute of Neurological Disorders and Stroke National Research Service Award Fellowship (F31NS129205 to S.S.‐K.). National Institutes of Health MARC U STAR undergraduate research program (T34GM062756 to J.W.).

## Ethics Statement

All mouse studies were done according to National Institutes of Health and Institutional Animal Care and Use Committee at the University of California Riverside (approval #20190015 and #20190029) guidelines; animal welfare assurance #A3439‐01 is on file with the Office of Laboratory Animal Welfare. Mice were maintained in an Association for Assessment and Accreditation of Laboratory Animal Care‐accredited facility under a 12 h light/dark cycle and fed standard mouse chow.

## Conflicts of Interest

The authors declare no conflicts of interest.

## Supporting information


**Figure S1:** Postnatal deletion of *Fmr1* from astrocytes affects synaptic and GABAergic gene expression in developing hippocampus of cKO mice.
**Table S1:** Differentially expressed genes analyzed with the NanoString “Glial Profiling” panel.
**Table S2:** vGAT/Gephyrin co‐localization via IHC image analysis & GABA_A_ receptor subunits' protein levels via western blot.
**Table S3:** Electrophysiology results.
**Table S4:** Electrophysiology results.
**Table S5:** GAT3 mRNA via qRT‐PCR and protein via western blot & GABA levels via IHC image analysis.
**Table S6:** PV levels and density via IHC image analysis.
**Table S7:** Behavioral test results.

## Data Availability

Supporting Information is available in the supplemental file and further data is available from the corresponding author upon request.
