# Supplementary material for: Hippocampal Astrocytes Impact Postnatal Development of Inhibitory Connections, Parvalbumin Levels, Social, and Spatial Navigation Behaviors in a Mouse Model of Fragile X Syndrome
Source: J Neurochem. 2026 Aug 23;170(8):e70541. doi: 10.1111/jnc.70541 (PMC13500873; doi:10.1111/jnc.70541)

Hippocampal astrocytes impact postnatal development of inhibitory connections, parvalbumin levels, social and spatial navigation behaviors in a mouse model of Fragile X Syndrome

Victoria A. Wagner[1,2#], Sarah Maples[3#], Ritika Thapa[1,2#], Jordan Wimberly[1], Ellie Pettijohn[1], Alexandra Varallo[1], Maham Rais[1], Samantha Sutley-Koury[1], Peter Hickmott[2,3], Iryna M. Ethell[1,2*]

[1] Division of Biomedical Sciences and Biomedical Sciences Graduate Program, University of California Riverside School of Medicine, Riverside, CA 92521

[2] Neuroscience Graduate Program, University of California Riverside, Riverside, CA, USA

[3]Department of Psychology, University of California Riverside, Riverside, CA, USA

[#] Co-first authors

*Corresponding Author:

Iryna M. Ethell, PhD

Division of Biomedical Sciences

Email: iryna.ethell@medsch.ucr.edu

Phone: 951-827-2186

Supplementary Materials

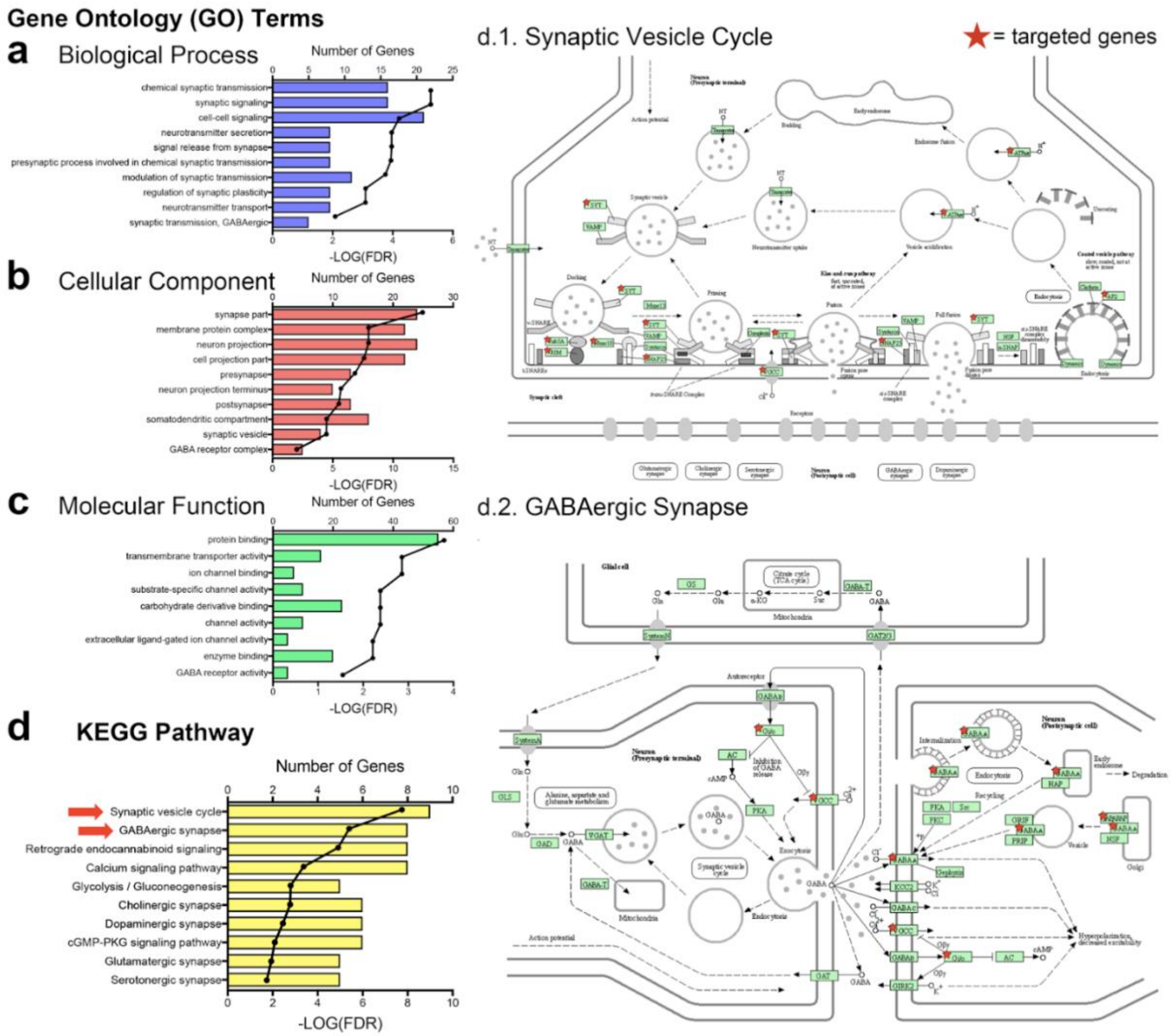

Supplementary Fig. 1 Postnatal deletion of *Fmr1* from astrocytes affects synaptic and GABAergic gene expression in developing hippocampus of cKO mice.

a-c, Functional enrichment of 81 DEGs using the Database for Annotation, Visualization and Integrated Discovery (DAVID) shows functional GO analysis of Biological Process (a), Cellular Component (b), and Molecular Function (c). At the level of Biological Process (a), the genes that were dysregulated due to *Fmr1* loss in astrocytes enriched heavily to the synaptic transmission GO terms, including: “synaptic signaling” (SNAP25, SYT4, GABRA1, RAB3A, SYT1, STXBP1, GABRA3, CACNA1B, GABRG2, GNAI1, P2RX7, RIMS1, SYNGR1,

KRAS, VDAC1, MAPT); “regulation of synaptic plasticity” (RIMS1, SNAP25, SYT4, SYNGR1, RAB3A, STXBP1, KRAS, MAPT); and “GABAergic synaptic transmission” (GABRA1, STXBP1, GABRG2). At the level of Cellular Component (b), the genes that were dysregulated due to *Fmr1* loss in astrocytes enriched heavily to the synapse part GO terms, including: “neuron projection” (SNAP25, CNTNAP2, SYT4, GABARAPL1, RAB3A, SYT1, STXBP1, ATP2B4, CACNA1B, FOS, GABRG2, SLC8A1, PPM1A, GRK3, SYNGR1, UCHL1, GNG3, GRIN3A, MAP2, GNB1, TIMP2, DMD, MAPT, AP2M1); “presynapse” (SNAP25, SYT4, RAB3A, SYT1, ABCC8, STXBP1, CACNA1B, P2RX7, RIMS1, SYNGR1, VDAC1, DNM1L, AP2M1); “postsynapse” (SYT4, GABRA1, GABRA3, GABRG2, SLC8A1, RIMS1, GRK3, GNG3, GRIN3A, ERBB3, MAP2, DMD, MAPT); and “GABA receptor complex” (GABRA1, GABRA3, GABRG2). At the level of Molecular Function (c), the genes that were dysregulated due to *Fmr1* loss in astrocytes enriched heavily to the protein binding GO terms, including: “transmembrane transporter activity” (SNAP25, GABRA1, ABCC8, GABRA3, ATP2B4, CACNA1B, AQP4, SLC2A5, ATP1B1, GABRG2, SLC8A1, P2RX7, GRIN3A, ATP6V1B2, VDAC1, ATP6V1D); “substrate-specific channel activity” (P2RX7, SNAP25, GABRA1, GRIN3A, ABCC8, GABRA3, CACNA1B, AQP4, VDAC1, GABRG2); “extracellular ligand-gated ion channel activity” (P2RX7, GABRA1, GRIN3A, GABRA3, GABRG2); and “GABA receptor activity” (GABRA1, GABRA3, GABRG2). Top x-axis represents number of DEGs mapping to the Gene Ontology (GO) term (FDR < 5%). **d**, DAVID Kyoto Encyclopedia of Genes and Genomes (KEGG) Pathway analysis. Red arrows indicate the top 2 pathways most closely associated with the 81 DEGs: Synaptic Vesicle Cycle and GABAergic Synapse. Additional pathway maps highlight the targeted genes (red stars) that were dysregulated due to *Fmr1* loss in astrocytes involved in the Synaptic Vesicle Cycle Pathway (d.1.) and GABAergic Synapse Pathway (d.2.).

Supplementary Table 1 – Differentially expressed genes analyzed with the NanoString "Glial Profiling" panel

Gene	Log2 fold change	std error (log2)	Linear fold change	Downregulated	P-value	method	probe.ID
Aldh1a1-mRNA	0.66	0.104	1.58		0.000712	loglinear	NM_013467.3:925
Dld-mRNA	-0.193	0.0305	0.875	1.142857	0.000731	loglinear	NM_007861.4:252
Serpina3n-mRNA	-2.9	0.467	0.134	7.462687	0.000809	lm.nb	NM_009252.2:119
Extl2-mRNA	0.332	0.057	1.26		0.00111	loglinear	NM_001163514.1:1052
Serpini1-mRNA	-0.822	0.154	0.566	1.766784	0.00176	lm.nb	NM_009250.2:968
Slc8a1-mRNA	-0.86	0.164	0.551	1.814882	0.00195	lm.nb	NM_011406.3:120
Slc25a4-mRNA	-0.266	0.051	0.831	1.203369	0.00197	loglinear	NM_007450.4:854
Pgam1-mRNA	-0.38	0.0737	0.768	1.302083	0.0021	loglinear	NM_023418.2:466
Prkar1a-mRNA	-0.277	0.0586	0.825	1.212121	0.00322	loglinear	NM_001313973.2:1270
Stab1-mRNA	0.404	0.0885	1.32		0.00383	loglinear	NM_138672.2:5890
Abcc8-mRNA	1.16	0.267	2.24		0.0048	lm.nb	NM_011510.3:1740
Psmd10-mRNA	-0.386	0.0893	0.765	1.30719	0.00497	loglinear	NM_001164177.1:215
Pdhb-mRNA	-0.295	0.0693	0.815	1.226994	0.00531	loglinear	NM_024221.3:845
Ndufa10-mRNA	-0.17	0.0417	0.889	1.124859	0.00644	loglinear	NM_024197.1:70
Sugt1-mRNA	-0.175	0.0433	0.886	1.128668	0.00682	loglinear	NM_026474.5:950
Ndufb5-mRNA	-0.486	0.121	0.714	1.40056	0.007	loglinear	NM_025316.2:106
Asns-mRNA	-0.412	0.103	0.752	1.329787	0.00709	loglinear	NM_012055.3:1606
Uchl1-mRNA	-0.534	0.134	0.691	1.447178	0.00717	lm.nb	NM_011670.2:278
Ppp2r2b-mRNA	-0.53	0.14	0.692	1.445087	0.00924	lm.nb	NM_028392.3:522

Dmd-mRNA	-0.448	0.121	0.733	1.364256	0.00986	loglinear	NM_007868.5:10045
Gabarapl1-mRNA	-0.22	0.0601	0.858	1.165501	0.0105	loglinear	NM_020590.4:420
Gng3-mRNA	-0.532	0.146	0.692	1.445087	0.0109	lm.nb	NM_010316.3:313
Stxbp1-mRNA	-0.416	0.114	0.749	1.335113	0.0109	lm.nb	NM_001113569.1:406
Mapt-mRNA	-0.162	0.0447	0.894	1.118568	0.0109	loglinear	NM_001038609.2:1202
Pgk1-mRNA	-0.225	0.0623	0.856	1.168224	0.0112	loglinear	NM_008828.2:507
Snap25-mRNA	-0.403	0.112	0.756	1.322751	0.0114	lm.nb	NM_011428.3:248
S100a10-mRNA	-0.817	0.228	0.568	1.760563	0.0117	lm.nb	NM_009112.2:154
Olfml3-mRNA	1.08	0.304	2.11		0.012	loglinear	NM_133859.2:1035
Ppm1a-mRNA	-0.178	0.0513	0.884	1.131222	0.0131	loglinear	NM_008910.3:602
Atp6v1b2-mRNA	-0.316	0.0911	0.803	1.24533	0.0133	loglinear	NM_007509.3:1017
Hspa1a/b-mRNA	0.837	0.244	1.79		0.0141	lm.nb	NM_010479.2:1819
Map2-mRNA	-0.591	0.175	0.664	1.506024	0.015	lm.nb	NM_008632.2:1462
Stambpl1-mRNA	-0.36	0.107	0.779	1.283697	0.0153	loglinear	NM_029682.4:1040
Gabra3-mRNA	-1.04	0.311	0.487	2.053388	0.0156	lm.nb	NM_008067.4:1485
Gabra1-mRNA	-0.521	0.158	0.697	1.43472	0.0164	lm.nb	NM_010250.4:905
Ppm1l-mRNA	-0.431	0.131	0.742	1.347709	0.0164	loglinear	NM_178726.3:1425
Fos-mRNA	-0.95	0.293	0.518	1.930502	0.0177	lm.nb	NM_010234.2:900
Rab3a-mRNA	-0.34	0.105	0.79	1.265823	0.018	lm.nb	NM_009001.6:288
Gnai1-mRNA	-0.366	0.115	0.776	1.28866	0.0192	lm.nb	NM_010305.1:470
Cacna1b-mRNA	-0.379	0.12	0.769	1.30039	0.0196	loglinear	NM_001042528.1:4345
Slc2a5-mRNA	-0.366	0.117	0.776	1.28866	0.0202	loglinear	NM_019741.3:760
Syngr1-mRNA	-0.358	0.114	0.78	1.282051	0.0204	loglinear	NM_009303.2:252

Fbxl3-mRNA	0.287	0.0919	1.22		0.0204	loglinear	NM_015822.2:724
Rbx1-mRNA	-0.228	0.0732	0.854	1.17096	0.0206	loglinear	NM_019712.3:185
P2rx7-mRNA	-0.367	0.119	0.776	1.28866	0.0214	loglinear	NM_001038839.2:378
Grk3-mRNA	-0.387	0.127	0.765	1.30719	0.0225	loglinear	NM_177078.4:424
Grin3a-mRNA	-0.789	0.262	0.579	1.727116	0.0235	lm.nb	NM_001033351.1:1332
Pdha1-mRNA	-0.221	0.0743	0.858	1.165501	0.0246	loglinear	NM_008810.3:440
Igfbp5-mRNA	0.431	0.145	1.35		0.025	lm.nb	NM_010518.2:1001
Kras-mRNA	-0.167	0.0585	0.891	1.122334	0.0291	loglinear	NM_021284.6:489
Phyh-mRNA	-0.216	0.076	0.861	1.16144	0.0294	loglinear	NM_010726.2:374
Syt4-mRNA	-0.338	0.119	0.791	1.264223	0.0298	loglinear	NM_009308.3:1120
Timp2-mRNA	-0.486	0.173	0.714	1.40056	0.0305	lm.nb	NM_011594.3:655
Atg12-mRNA	-0.182	0.0648	0.881	1.135074	0.0307	loglinear	NM_026217.3:390
Fcrls-mRNA	0.622	0.222	1.54	0.649351	0.0311	lm.nb	NM_030707.3:925
Map3k4-mRNA	0.46	0.164	1.38		0.0312	lm.nb	NM_011948.2:2138
Rab6b-mRNA	-0.235	0.0844	0.85	1.176471	0.0318	loglinear	NM_173781.4:505
Atp5pb-mRNA	-0.192	0.069	0.875	1.142857	0.0318	loglinear	NM_009725.3:704
Acsl4-mRNA	-0.357	0.129	0.781	1.28041	0.0323	lm.nb	NM_207625.2:625
Ccr5-mRNA	1.23	0.451	2.34		0.0343	lm.nb	NM_009917.5:1018
Tardbp-mRNA	0.121	0.0447	1.09		0.0349	loglinear	NM_001008545.2:825
Gabrg2-mRNA	-0.46	0.17	0.727	1.375516	0.0354	lm.nb	NM_177408.5:1140
Atp6v1d-mRNA	-0.286	0.107	0.82	1.219512	0.0368	lm.nb	NM_023721.2:408
Syt1-mRNA	-0.352	0.131	0.784	1.27551	0.0368	lm.nb	NM_009306.3:711
Ap2m1-mRNA	-0.217	0.0817	0.86	1.162791	0.0375	loglinear	NM_009679.2:1294

Rims1-mRNA	-0.359	0.136	0.78	1.282051	0.0382	lm.nb	NM_053270.1:2220
Pde1a-mRNA	-0.718	0.273	0.608	1.644737	0.0391	lm.nb	NM_001159582.1:1165
Meaf6-mRNA	-0.261	0.0994	0.835	1.197605	0.0392	loglinear	NM_027310.3:178
Ccnh-mRNA	-0.184	0.0702	0.88	1.136364	0.0397	loglinear	NM_023243.2:960
Aqp4-mRNA	0.455	0.174	1.37		0.0399	lm.nb	NM_009700.2:130
Atp2b4-mRNA	-0.895	0.343	0.538	1.858736	0.0401	lm.nb	NM_001167949.1:3245
Eloc-mRNA	-0.152	0.0584	0.9	1.111111	0.0406	loglinear	NM_001310470.1:492
Vdac1-mRNA	-0.223	0.0862	0.857	1.166861	0.0412	loglinear	NM_011694.4:562
Dnm1l-mRNA	-0.16	0.0628	0.895	1.117318	0.0432	loglinear	NM_001025947.1:2075
Atp1b1-mRNA	-0.286	0.112	0.82	1.219512	0.0434	lm.nb	NM_009721.6:1018
Cntnap2-mRNA	-0.563	0.221	0.677	1.477105	0.0437	lm.nb	NM_001004357.2:3985
Erbb3-mRNA	1.27	0.505	2.42		0.0454	lm.nb	NM_010153.1:1290
Nes-mRNA	0.45	0.18	1.37		0.0461	loglinear	NM_016701.3:2716
Sirpa-mRNA	-0.313	0.126	0.805	1.242236	0.0477	lm.nb	NM_001291020.1:1613
Gnb1-mRNA	-0.203	0.0824	0.869	1.150748	0.0492	loglinear	NM_008142.3:311
Ubqln2-mRNA	-0.229	0.0931	0.854	1.17096	0.0495	loglinear	NM_018798.2:1970

List of primer sequences used for qRT-PCR

Gene	Forward primer sequence (5'–3')	Reverse primer sequence (5'–3')
Fmr1 (exon 16/17)	CCGAACAGATAATCGTCCACG	ACGCTGTCTGGCTTTTCCTTC
PV	GGCCTGAAGAAAAAGAACCCG	ATCTTGCCGTCCCCATCCTT
Slc6a11	CGG CTGGGTATATGGAAGCA	ACGACTTTCCAGCACCACTT
GAPDH	ACTCCACTCACGGCAAATTC	TCTCCATGGTGGTGAAGACA

Fmr1 mRNA	**Mean**	**SEM**	**N**
Ctrl WT	1.001	0.02135	5

cKO	0.8659	0.01333	4
Statistics	Two-tailed, unpaired t-test	t=5.024, df=7, p= 0.0015 η^2= 0.7829	

FMRP in tdTomato-positive astrocytes

**Shapiro-Wilk Test**	Ctrl WT	cKO
W	0.9141	0.6557
P value	0.0762	<0.0001
Passed normality test (alpha =0.05)?	Yes	No
P value summary	ns	****

**Mann Whitney Test**	
P value	0.0001
Exact or approximate P value?	Exact
P value summary	***
Significant different (p< 0.05)?	Yes
One- or two- tailed P value?	Two-tailed
Sum of ranks in column A,B	499, 204
Mann-Whitney U	51

FMRP in astrocytes – GABA GS

**Shapiro-Wilk Test**	Ctrl WT	cKO
W	0.9115	0.8353
P value	0.2231	0.0243
Passed normality test (alpha =0.05)?	Yes	No
P value summary	Ns	*

**Mann Whitney Test**	
P value	0.0325
Exact or approximate P value?	Exact
P value summary	*
Significant different (p< 0.05)?	Yes
One- or two- tailed P value?	Two-tailed
Sum of ranks in column A,B	187, 113
Mann-Whitney U	35

Gabra1 mRNA

**Shapiro-Wilk Test**	Ctrl WT	cKO
W	0.8309	0.9192
P value	0.1701	0.5326
Passed normality test (alpha =0.05)?	Yes	Yes
P value summary	Ns	ns

Gabra1 mRNA	**Mean**	**SEM**	**N**
Ctrl WT	1.000	0.0883	4
cKO	0.7260	0.063529	4
Statistics	Two-tailed, unpaired t-test	t=2.519, df=6, p= 0.0454 η^2= 0.5140	

Gabra3 mRNA

**Shapiro-Wilk Test**	Ctrl WT	cKO
W	0.9224	0.8506
P value	0.5503	0.2280
Passed normality test (alpha =0.05)?	Yes	Yes
P value summary	ns	ns

Gabra3 mRNA	**Mean**	**SEM**	**N**
Ctrl WT	1.000	0.185032	4
cKO	0.5370	0.087587	4
Statistics	Two-tailed, unpaired t-test	t=2.262, df=6, p= 0.0644 η^2= 0.4602	

Gabra5 mRNA

**Shapiro-Wilk Test**	Ctrl WT	cKO
W	0.7707	0.7824
P value	0.0592	0.0742
Passed normality test (alpha =0.05)?	Yes	Yes
P value summary	ns	ns

Gabra5 mRNA	**Mean**	**SEM**	**N**
Ctrl WT	1.000	0.233494	4
cKO	0.7260	0.230942	4
Statistics	Two-tailed, unpaired t-test	t=0.5109, df=6, p= 0.6277 η^2= 0.04169	

Gabrg2 mRNA

**Shapiro-Wilk Test**	Ctrl WT	cKO
W	0.9455	0.8858
P value	0.6882	0.3638
Passed normality test (alpha =0.05)?	Yes	Yes
P value summary	ns	ns

Gabrg2 mRNA	**Mean**	**SEM**	**N**
Ctrl WT	1.000	0.057681	4

cKO	0.7480	0.084202	4
Statistics	Two-tailed, unpaired t-test	t=2.469, df=6, p= 0.0485<br>η^2= 0.5040	

Supplementary Table 2 - vGAT/Gephyrin co-localization via IHC image analysis & GABA$_A$ receptor subunits' protein levels via Western Blot

**Shapiro-Wilk Test**	Ctrl WT	cKO
W	0.9712	0.9760
P value	0.4956	0.8069
Passed normality test (alpha =0.05)?	Yes	Yes
P value summary	ns	ns

vGAT/Gephyrin co-localization	**Mean**	**SEM**	**N**
Ctrl WT	59.62	1.718	34 images
cKO	51.78	3.176	31 images
Statistics	Two-tailed, unpaired t-test	t=2.224, df=63, p= 0.0297<br>η^2= 0.07280	

vGAT vGAT/Gephyrin co-localization	**Min**	**Max**	**Median**	**Q1**	**Q3**	**IQR**
Ctrl WT	37.096	88.087	59.623	53.968	65.401	11.433
cKO	19.954	87.512	49.868	39.717	62.266	22.549

vGAT puncta density

**Shapiro-Wilk Test**	Ctrl WT	cKO
W	0.9614	0.9615
P value	0.2674	0.3380
Passed normality test (alpha =0.05)?	Yes	Yes
P value summary	ns	ns

vGAT puncta density	**Mean**	**SEM**	**N**
Ctrl WT	83.78	2.620	34 images
cKO	77.13	4.378	30 images
Statistics	Two-tailed, unpaired t-test	t=1.339, df=62, p= 0.1853<br>η^2= 0.02812	

vGAT puncta density	**Min**	**Max**	**Median**	**Q1**	**Q3**	**IQR**
Ctrl WT	45.587	119.262	80.9065	75.412	95.09	19.678
cKO	33.353	117.689	71.218	60.182	100.413	40.231

GABAARγ2 protein

**Shapiro-Wilk Test**	Ctrl WT	cKO
W	1.000	0.9735
P value	>0.9999	0.8630
Passed normality test (alpha =0.05)?	Yes	Yes
P value summary	ns	ns

GABAARγ2 protein	**Mean**	**SEM**	**N**
Ctrl WT	1.000	0.01905	3
cKO	0.6371	0.08447	4
Statistics	Two-tailed, unpaired t-test	t=3.585, df=5, p= 0.0158 η^2= 0.7200	

GABAARα5 protein

**Shapiro-Wilk Test**	Ctrl WT	cKO
W	0.9094	0.9143
P value	0.4791	0.5054
Passed normality test (alpha =0.05)?	Yes	Yes
P value summary	ns	ns

GABAARα5 protein	**Mean**	**SEM**	**N**
Ctrl WT	0.9999	0.09511	4
cKO	1.039	0.2490	4
Statistics	Two-tailed, unpaired t-test	t=0.1477, df=6, p= 0.8874 η^2= 0.003624	

Supplementary Table 3 – Electrophysiology results

sIPSC amplitude WT vs tamoxifen-injected Ctrl WT

**Shapiro-Wilk Test**	WT	Ctrl WT
W	0.9599	0.7733
P value	0.7845	0.0101
Passed normality test (alpha =0.05)?	Yes	No
P value summary	ns	*

n	10	9

Mann Whitney Test	
P value	0.2344
Exact or approximate P value?	Exact
P value summary	ns
Significant different (p< 0.05)?	No
One- or two- tailed P value?	Two-tailed
Sum of ranks in column A,B	85, 105
Mann-Whitney U	30

sIPSC frequency WT vs tamoxifen-injected Ctrl WT

Shapiro-Wilk Test	WT	Ctrl WT
W	0.8633	0.7723
P value	0.1042	0.0067
Passed normality test (alpha =0.05)?	Yes	No
P value summary	ns	**
n	9	10

Mann Whitney Test	
P value	0.6176
Exact or approximate P value?	Exact
P value summary	ns
Significant different (p< 0.05)?	No
One- or two- tailed P value?	Two-tailed
Sum of ranks in column A,B	96.50, 93.50
Mann-Whitney U	38.50

Tonic GABA

Shapiro-Wilk Test	WT	Ctrl WT
W	0.8731	0.9699
P value	0.1616	0.8852
Passed normality test (alpha =0.05)?	Yes	Yes
P value summary	ns	ns
n	8	11

Mann Whitney Test	
P value	0.9678

Exact or approximate P value?	Exact
P value summary	ns
Significant different (p< 0.05)?	No
One- or two- tailed P value?	Two-tailed
Sum of ranks in column A,B	81, 109
Mann-Whitney U	43

sIPSC amplitude

**Shapiro-Wilk Test**	WT	KO	cKO
W	0.9561	0.8529	0.8389
P value	0.4978	0.0466	0.0269
Passed normality test (alpha =0.05)?	Yes	No	No
P value summary	ns	*	*
n	19	11	12

**Kruskal-Wallis Test**	
P value	0.0037
Exact or approximate P value?	Approximate
P value summary	**
Do the median vary signif. (p< 0.05)?	Yes
Number of groups	3
Kruskal-Wallis stastic	11.21

**Dunn's multiple comparisons test**	**Mean Diff.**	**Significant?**	**Adjusted P Value**
WT vs. Global KO	-12.66	Yes	0.0129
WT vs. cKO	-12.81	Yes	0.0093

sIPSC frequency

**Shapiro-Wilk Test**	WT	KO	cKO
W	0.8023	0.9596	0.9171
P value	0.0012	0.7814	0.2627
Passed normality test (alpha =0.05)?	No	Yes	Yes
P value summary	**	ns	ns
n	19	10	12

**Kruskal-Wallis Test**	
P value	0.8189
Exact or approximate P value?	Approximate

P value summary	ns
Do the median vary signif. (p< 0.05)?	No
Number of groups	3
Kruskal-Wallis stastic	0.3997

Supplementary Table 4 – Electrophysiology results

Tonic inhibition

**Shapiro-Wilk Test**	WT	KO	cKO
W	0.9115	0.9455	0.7741
P value	0.0787	0.5322	0.0017
Passed normality test (alpha =0.05)?	Yes	Yes	No
P value summary	ns	ns	**
n	19	13	15

**Kruskal-Wallis test**	**P value**	**Number of groups**	**Kruskal-Wallis statistic**
Genotype	0.0206	3	7.763

**Dunn's multiple comparisons test**	**Mean rank diff.**	**Adjusted P Value**
WT vs. Global KO	12.74	0.0197
WT vs. cKO	0.6702	>0.9999

Tonic inhibition – SNAP

**Shapiro-Wilk Test**	WT SNAP	KO SNAP	cKO SNAP
W	0.8998	0.9109	0.8516
P value	0.0944	0.3607	0.0607
Passed normality test (alpha =0.05)?	Yes	Yes	Yes
P value summary	ns	ns	ns
n	15	8	10

**Brown-Forsythe ANOVA test**	**F* (DFn, DFd)**	**P value**
Genotype	7.076 (2.000, 23.71)	0.0039

**Dunn's multiple comparisons test**	**Mean diff.**	**95.00% CI of diff.**	**Adjusted P Value**
WT vs. Global KO	19.43	4.425 to 34.44	0.0112

WT vs. cKO	-10.72	-31.33 to 9.894	0.3948

Supplementary Table 5 – GAT3 mRNA via qRT-PCR and protein via western blot & GABA levels via IHC image analysis

GAT3 mRNA

**Shapiro-Wilk Test**	Ctrl WT	cKO
W	0.9712	0.9699
P value	0.8829	0.8411
Passed normality test (alpha =0.05)?	Yes	Yes
P value summary	ns	ns

GAT3 mRNA	**Mean**	**SEM**	**N**
Ctrl WT	1.061	0.1633	5
cKO	0.8983	0.1016	4
Statistics	Two-tailed, unpaired t-test	t=0.7896, df=7, p=0.4557 η^2= 0.08178	

GAT3 Protein

**Shapiro-Wilk Test**	Ctrl WT	cKO
W	0.9700	0.6669
P value	0.6678	0.0044
Passed normality test (alpha =0.05)?	Yes	No
P value summary	ns	**
n	3	4

GAT3 protein	**Mean**	**SEM**	**N**
Ctrl WT	1.000	0.1537	3
cKO	0.6132	0.06560	4
Statistics	Two-tailed, unpaired t-test	t=2.576, df=5, p=0.0496 η^2= 0.5704	

GABA levels

**Shapiro-Wilk Test**	WT - veh	cKO - veh	WT - SNAP	cKO - SNAP
W	0.8953	0.8458	0.9118	0.9318
P value	0.0476	0.0073	0.0927	0.2088
Passed normality test (alpha =0.05)?	No	No	Yes	Yes
P value summary	*	**	ns	ns

**ANOVA table**	**SS (Type III)**	**DF**	**MS**	**F (DFn, DFd)**	**P value**	**% of total variation**

Interaction	9105	1	9105	F (1, 68) = 16.00	P=0.0002	9.691
Genotype	6153	1	6153	F (1, 68) = 10.81	P=0.0016	6.549
Treatment	40005	1	40005	F (1, 68) = 70.31	P<0.0001	42.58

Uncorrected Fisher's LSD	Mean diff.	95.00% CI of diff.	Individual P Value
Ctrl WT			
Vehicle vs. SNAP	-24.65	-40.52 to -8.786	0.0028
cKO			
Vehicle vs. Vehicle	-69.63	-85.50 to -53.77	<0.0001
Vehicle			
Ctrl WT vs. cKO	4.002	-11.86 to 19.87	0.6164
SNAP			
Ctrl WT vs. cKO	-40.98	-56.85 to -25.11	<0.0001

Whisker description

GABA levels	**Min**	**Max**	**Median**	**Q1**	**Q3**	**IQR**
Ctrl WT	18.719	60.258	45.825	27.377	52.052	24.676
cKO	18.414	62.582	39.677	19.353	52.700	33.347
Ctrl WT SNAP	26.465	108.660	65.499	37.16	87.038	49.878
cKO SNAP	52.453	191.202	108.524	90.266	120.816	30.550

Supplementary Table 6 – PV levels and density via IHC image analysis

PV levels

**Shapiro-Wilk Test**	WT - veh	cKO - veh	WT - SNAP	cKO - SNAP
W	0.7583	0.9506	0.9286	0.8586
P value	0.0008	0.6461	0.3655	0.0470
Passed normality test (alpha =0.05)?	No	Yes	Yes	No
P value summary	***	ns	ns	*
n	16	12	12	12

ANOVA table	SS (Type III)	DF	MS	F (DFn, DFd)	P value	% of total variation
Interaction	13162	1	13162	F (1, 48) = 4.596	P=0.0371	7.325
Genotype	4392	1	4392	F (1, 48) = 1.533	P=0.2216	2.444
Treatment	26483	1	26483	F (1, 48) = 9.247	P=0.0038	42.58

Uncorrected Fisher's LSD	Predicted (LS) mean diff.	95.00% CI of diff.	Adjusted P Value

WT :Vehicle vs. WT :SNAP	-13.42	-54.51 to 27.67	0.5146
WT :Vehicle vs. KO :Vehicle	13.54	-27.55 to 54.64	0.5107
WT :SNAP vs. KO :SNAP	-50.59	-94.52 to -6.662	0.0249
KO :Vehicle vs. KO :SNAP	-77.55	-121.5 to -33.62	0.0009

PV cell density

**Shapiro-Wilk Test**	WT - veh	cKO - veh	WT - SNAP	cKO - SNAP
W	0.9549	0.9510	0.8786	0.9530
P value	0.4471	0.6516	0.0167	0.6808
Passed normality test (alpha =0.05)?	Yes	Yes	No	Yes
P value summary	ns	ns	*	ns
n	20	12	20	12

**ANOVA table**	**SS (Type III)**	**DF**	**MS**	**F (DFn, DFd)**	**P value**	**% of total variation**
Interaction	0.002937	1	0.002937	F (1, 60) = 0.1383	P=0.7113	0.2061
Genotype	0.02030	1	0.02030	F (1, 60) = 0.9561	P=0.3321	1.425
Treatment	0.1106	1	0.1106	F (1, 60) = 5.210	P=0.0260	7.763

**Tukey's multiple comparisons test**	**Predicted (LS) mean diff.**	**95.00% CI of diff.**	**Adjusted P Value**
Ctrl WT :Vehicle vs. Ctrl WT :SNAP	0.09988	-0.02189 to 0.2216	0.1442
Ctrl WT :Vehicle vs. cKO:Vehicle	-0.02280	-0.1634 to 0.1178	0.9734
Ctrl WT :Vehicle vs. cKO:SNAP	0.04909	-0.09151 to 0.1897	0.7929
Ctrl WT :SNAP vs. cKO:Vehicle	-0.1227	-0.2633 to 0.01794	0.1082
Ctrl WT :SNAP vs. cKO:SNAP	-0.05078	-0.1914 to 0.08982	0.7756
cKO:Vehicle vs. cKO:SNAP	0.07189	-0.08532 to 0.2291	0.6240

Supplementary Table 7 – Behavioral test results

Social preference 0-5 min

**Shapiro-Wilk Test**	WT – Empty	WT – S1	cKO – Empty	cKO – S1
W	0.9699	0.9771	0.9405	0.9495
P value	0.5570	0.7592	0.2453	0.3603

Passed normality test (alpha =0.05)?	Yes	Yes	Yes	Yes
P value summary	ns	ns	ns	ns
n	29	29	20	20

**ANOVA table**	**SS (Type III)**	**DF**	**MS**	**F (DFn, DFd)**	**P value**	**% of total variation**
Stranger x Genotype	42.61	1	42.61	F (1, 47) = 0.1731	P=0.6793	0.2046
Stranger	3268	1	3268	F (1, 47) = 13.27	P=0.0007	15.69
Genotype	5835	1	136.0	F (1, 47) = 1.095	P=0.3007	0.6530

**Bonferroni's multiple comparisons test**	**Predicted (LS) mean diff.**	**95.00% CI of diff.**	**Adjusted P Value**
Empty			
WT vs. cKO	-1.055	-10.06 to 7.594	>0.9999
S1			
WT vs. cKO	-3.738	-12.75 to 5.271	0.6940
WT			
Empty vs. S1	-10.41	-19.95 to -0.8667	0.0299
cKO			
Empty vs. S1	-13.09	-24.58 to -1.602	0.0225

Whisker description

Social Preference – no treatment	**Min**	**Max**	**Median**	**Q1**	**Q3**	**IQR**
Ctrl WT – E	3.2	61	24.4	13.6	35.05	21.45
Ctrl WT – S1	8.3	74.3	33.5	25.3	46.65	21.35
cKO - E	11.9	52	25.35	18.15	33.9	15.75
cKO – S1	11.2	55.9	40.2	29.8	50.75	20.95

Social preference 0-5 min treatment

**Shapiro-Wilk Test**	cKO Veh - E	cKO Veh – S1	cKO SNAP – veh	cKO SNAP– S1
W	0.7975	0.8775	0.9311	0.9774
P value	0.0046	0.0536	0.4590	0.9496
Passed normality test (alpha =0.05)?	No	Yes	Yes	Yes
P value summary	**	ns	ns	ns

n	13	13	10	10

ANOVA table	SS	DF	MS	F (DFn, DFd)	P value	% of total variation
Stranger x Treatment	81.05	1	81.05	F (1, 21) = 0.2143	P=0.6482	0.4950
Stranger	6806	1	6806	F (1, 21) = 18.00	P=0.0004	41.57
Treatment	8.556	1	8.556	F (1, 21) = 0.1474	P=0.7049	0.5226

Bonferroni's multiple comparisons test	Predicted (LS) mean diff.	95.00% CI of diff.	Adjusted P Value
Empty			
cKO Veh vs. cKO SNAP	-1.808	-16.25 to 12.63	>0.9999
S1			
cKO Veh vs. cKO SNAP	3.548	-10.89 to 17.99	>0.9999
cKO Veh			
Empty vs. S1	-27.22	-45.63 to -8.802	0.0036
cKO SNAP			
Empty vs. S1	-21.86	-42.85 to -0.8656	0.0404

Whisker description

	Min	Max	Median	Q1	Q3	IQR
cKO Veh - E	8.6	55.2	20.7	14.35	27.7	13.35
cKO Veh – S1	9	70	55.1	41.15	62.1	20.95
cKO SNAP - E	11.7	45.6	24	19.4	27.3	7.9
cKO SNAP – S1	20	77.8	47.45	35	56	21

Social Novelty Preference

Shapiro-Wilk Test	WT – S1	WT – S2	cKO – S1	cKO – S2
W	0.9739	0.9175	0.9415	0.9671
P value	0.6495	0.0231	0.1939	0.6198
Passed normality test (alpha =0.05)?	Yes	Yes	Yes	Yes
P value summary	ns	ns	ns	ns
n	29	29	22	22

ANOVA table	SS (Type III)	DF	MS	F (DFn, DFd)	P value	% of total variation
Stranger x Genotype	100.2	1	100.2	F (1, 49) = 0.4854	P=0.4893	0.665
Stranger	2448	1	2448	F (1, 49) = 11.86	P=0.0012	16.29
Genotype	11.36	1	11.36	F (1, 49) = 0.2565	P=0.6148	0.07558

Bonferroni's multiple comparisons test	Predicted (LS) mean diff.	95.00% CI of diff.	Adjusted P Value
S1			
WT vs. cKO	-2.675	-9.881 to 4.531	0.8003
S2			
WT vs. cKO	1.327	-5.878 to 8.533	>0.9999
WT			
S1 vs. S2	-11.89	-20.62 to -3.169	0.0055
cKO			
S1 vs. S2	-7.891	-17.91 to 2.126	0.1492

Whisker description

Social Novelty Preference – no treatment	**Min**	**Max**	**Median**	**Q1**	**Q3**	**IQR**
Ctrl WT – S1	11.6	53.7	25.6	19.4	33.55	14.15
Ctrl WT – S2	18.1	58.6	40.3	31	47.1	16.1
cKO – S1	14.4	48.3	27.15	21	38.8	17.8
cKO – S2	14.4	60.1	38.3	28.4	50.6	22.2

Social Novelty Preference- treatment

**Shapiro-Wilk Test**	cKO Veh – S1	cKO Veh – S2	cKO SNAP – S1	cKO SNAP – S2
W	0.8563	0.8822	0.9311	0.9774
P value	0.0345	0.0764	0.4590	0.9496
Passed normality test (alpha =0.05)?	No	Yes	Yes	Yes
P value summary	*	ns	ns	ns
n	13	13	10	10

ANOVA table	SS (Type I II)	DF	MS	F (DFn, DFd)	P value	% of total variation
Stranger x Treatment	105.6	1	105.6	F (1, 22) = 0.5767	P=0.4557	1.207
Stranger	1859	1	1859	F (1, 22) = 10.16	P=0.0043	21.25
Treatment	33.41	1	33.41	F (1, 22) = 0.2608	P=0.6147	0.3819

Bonferroni's multiple comparisons test	Predicted (LS) mean diff.	95.00% CI of diff.	Adjusted P Value

S1			
cKO Veh vs. cKO SNAP	1.316	-10.67 to 13.30	>0.9999
S2			
cKO Veh vs. cKO SNAP	-4.700	-16.68 to 7.284	0.7354
cKO Veh			
S1 vs. S2	-9.614	-21.91 to 2.686	0.1468
cKO SNAP			
S1 vs. S2	-15.63	-30.18 to -1.077	0.0339

Whisker description

Social Novelty Preference – no treatment	**Min**	**Max**	**Median**	**Q1**	**Q3**	**IQR**
cKO Veh – S1	8.7	55.1	22.25	18.9	39.5	20.6
cKO Veh – S2	9.6	63.5	39.1	31.8	41.1	9.3
cKO SNAP – S1	15	39.8	24.35	21.4	27.8	6.4
cKO SNAP – S2	20.2	56.1	45.4	30.5	52.4	21.9

Object Location Memory Test: WT vs cKO (untreated)

**Shapiro-Wilk Test**	WT – O1	WT – O2	cKO – O1	cKO – O2
W	0.9416	0.9181	0.9491	0.9415
P value	0.6267	0.4545	0.7018	0.6254
Passed normality test (alpha =0.05)?	Yes	Yes	Yes	Yes
P value summary	ns	ns	ns	ns
n	8	7	8	8

**Mixed-effects analysis**	**F (DFn, DFd)**	**P value**
Object	F (1, 8) = 29.94	P=0.0006
Genotype	F (1, 8) = 1.225	P=0.3006
Object x Genotype	F (1, 3) = 2.997	P=0.1819

**Tukey's multiple comparisons test**	**Predicted (LS) mean diff.**	**95.00% CI of diff.**	**Adjusted P Value**
O1:WT vs. O1:KO	-0.01466	-0.2184 to 0.1891	0.9831
O1:WT vs. O2:WT	-0.2077	-0.4089 to -0.006466	0.0460
O1:WT vs. O2:KO	-0.1225	-0.3262 to 0.08123	0.1728
O1:KO vs. O2:WT	-0.1930	-0.4038 to 0.01781	0.0630

O1:KO vs. O2:KO	-0.1078	-0.3001 to 0.08437	0.2004
O2:WT vs. O2:KO	0.08515	-0.1257 to 0.2960	0.3688

Object Location Preference	**Min**	**Max**	**Median**	**Q1**	**Q3**	**IQR**
Ctrl WT – O1	0.332	0.489	0.426	0.391	0.475	0.083
Ctrl WT – O2	0.579	0.714	0.609	0.594	0.668	0.073
cKO – O1	0.345	0.526	0.431	0.400	0.495	0.094
cKO – O2	0.338	0.727	0.526	0.460	0.676	0.217

Object Location Memory Test: cKO veh vs cKO SNAP

**Shapiro-Wilk Test**	cKO Veh – O1	cKO Veh – O2	cKO SNAP– O1	cKO SNAP – O2
W	0.9813	0.8739	0.8919	0.9463
P value	0.9692	0.1646	0.1037	0.5430
Passed normality test (alpha =0.05)?	Yes	Yes	Yes	Yes
P value summary	ns	ns	ns	ns
n	8	8	13	13

**Mixed-effects analysis**	**F (DFn, DFd)**	**P value**
Object x Treatment	F (1, 38) = 3.693	P=0.0621
Object	F (1, 38) = 0.008622	P=0.9265
Treatment	F (1, 38) = 1.796	P=0.1882

**Uncorrected Fisher's LSD**	**Predicted (LS) mean diff.**	**95.00% CI of diff.**	**Individual P Value**
O1			
KO veh vs. KO SNAP	0.04406	-0.05708 to 0.1452	0.3834
O2			
KO veh vs. KO SNAP	-0.05062	-0.1518 to 0.05052	0.3174
KO veh			
O1 vs. O2	-0.02056	-0.1331 to 0.09199	0.7136
KO SNAP			
O1 vs. O2	-0.1152	-0.2035 to -0.02695	0.0119

Whisker description

Object Location Preference	**Min**	**Max**	**Median**	**Q1**	**Q3**	**IQR**
cKO Veh – O1	0.406	0.595	0.492	0.463	0.534	0.072
cKO Veh– O2	0.418	0.573	0.518	0.507	0.553	0.046
cKO SNAP– O1	0.216	0.757	0.449	0.396	0.473	0.076
cKO SNAP – O2	0.407	0.795	0.577	0.479	0.691	0.211

Western blots

GABRg2 blot for Figure 2c

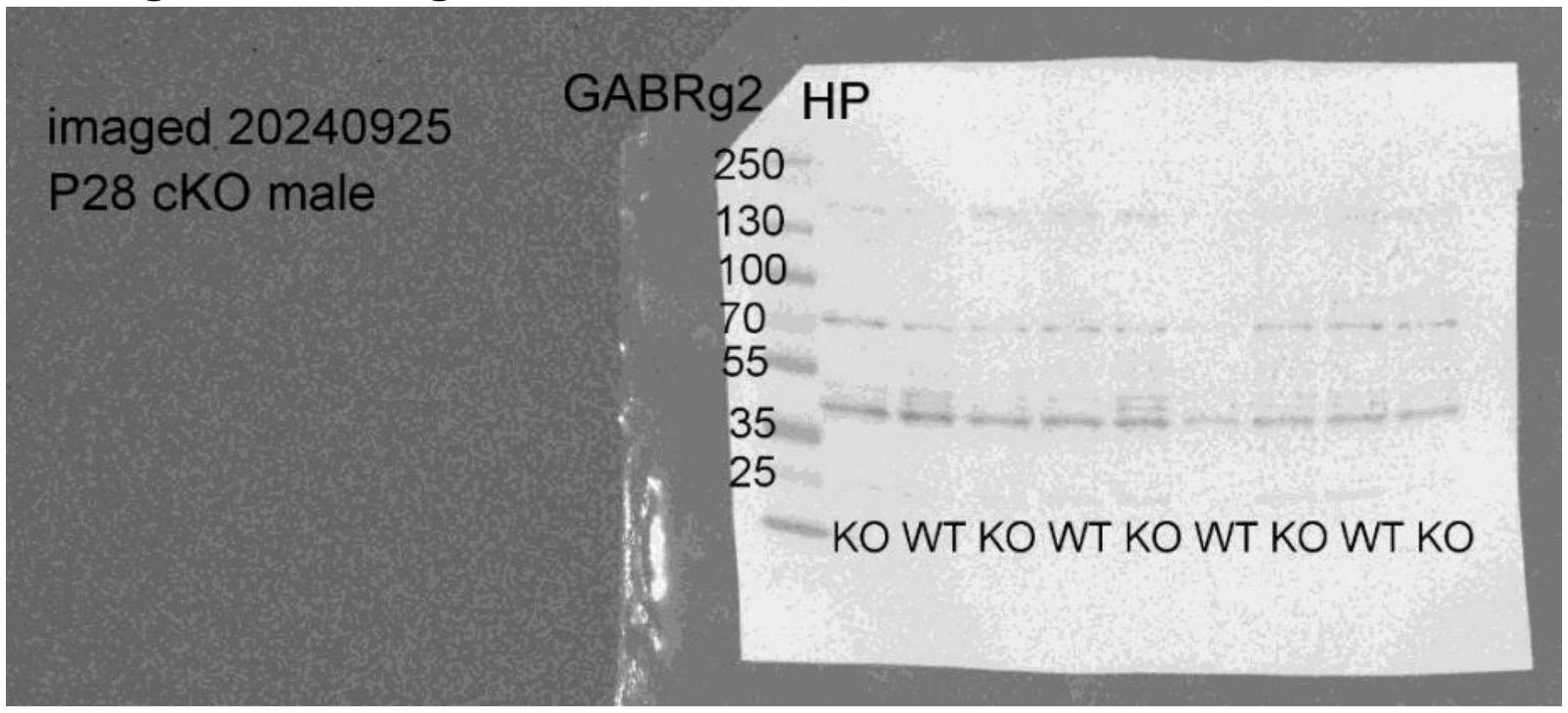

Beta-actin blot for GABRg2 for Figure 2c (same blot as shown in Figure 6d)

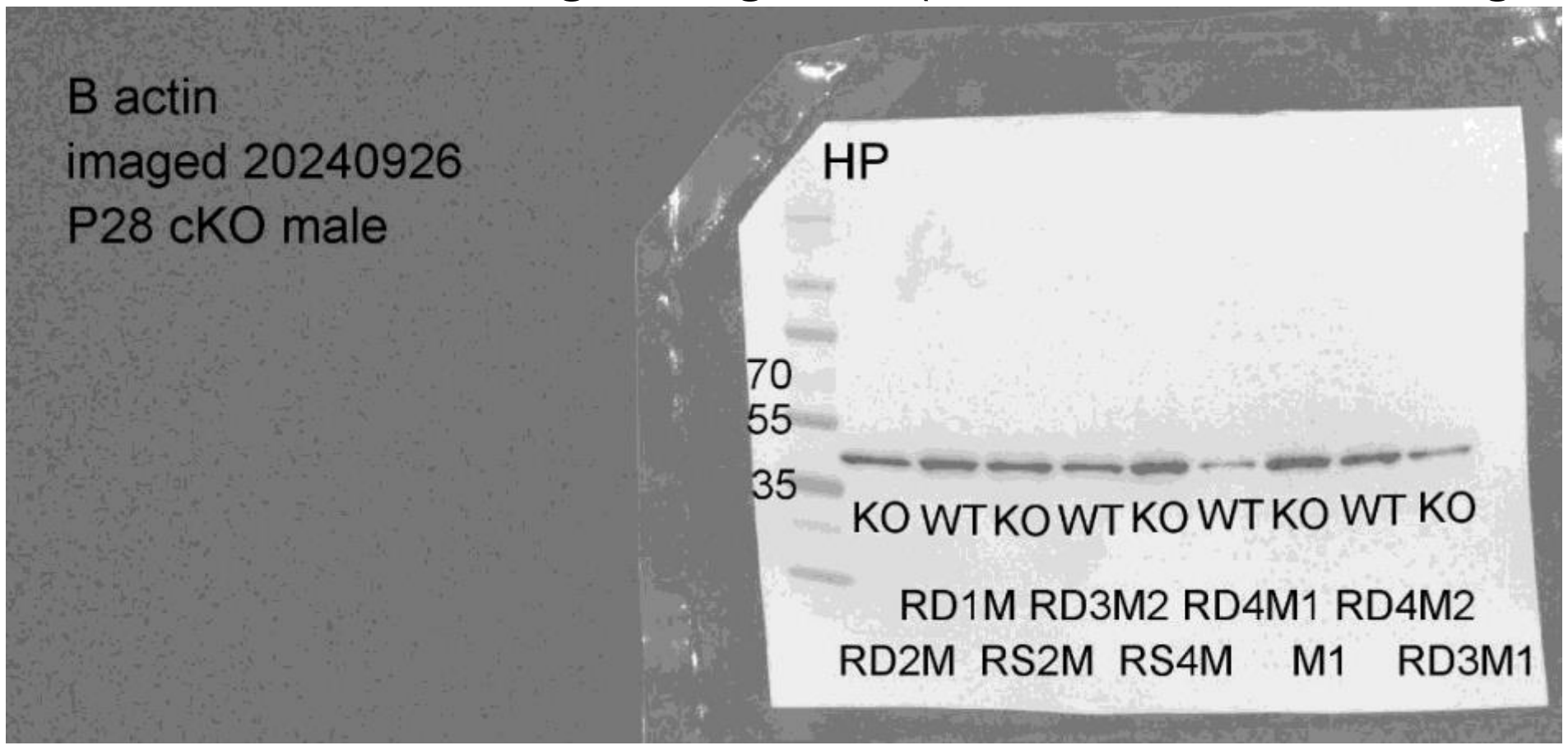

GABRa5 blot for Figure 2c

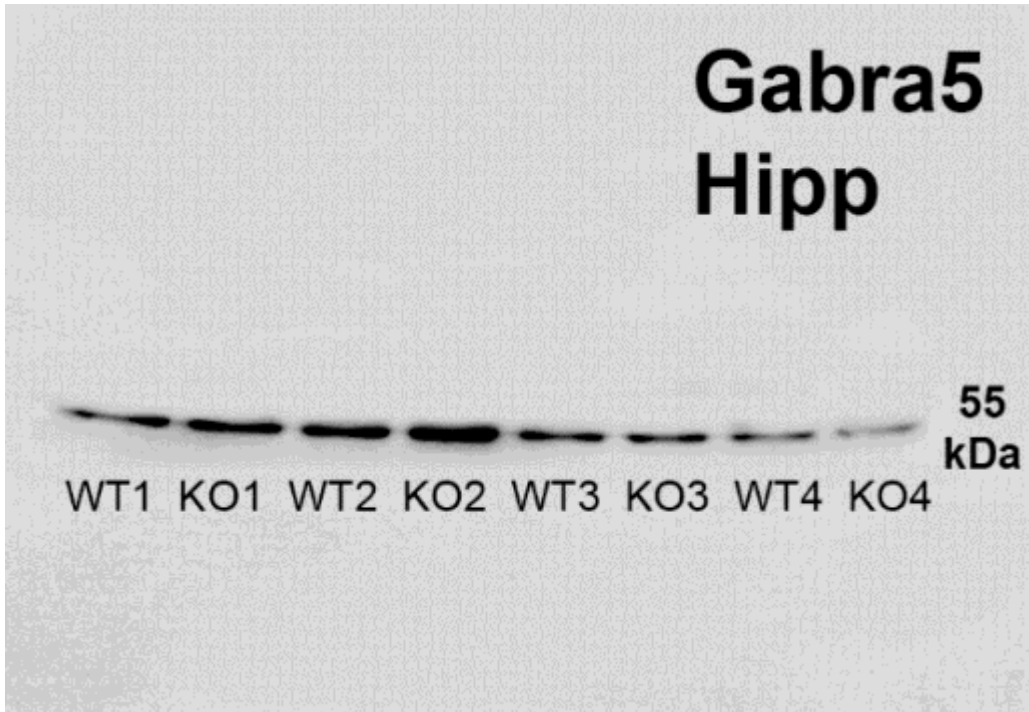

Beta-actin blot for GABRa5 for Figure 2c

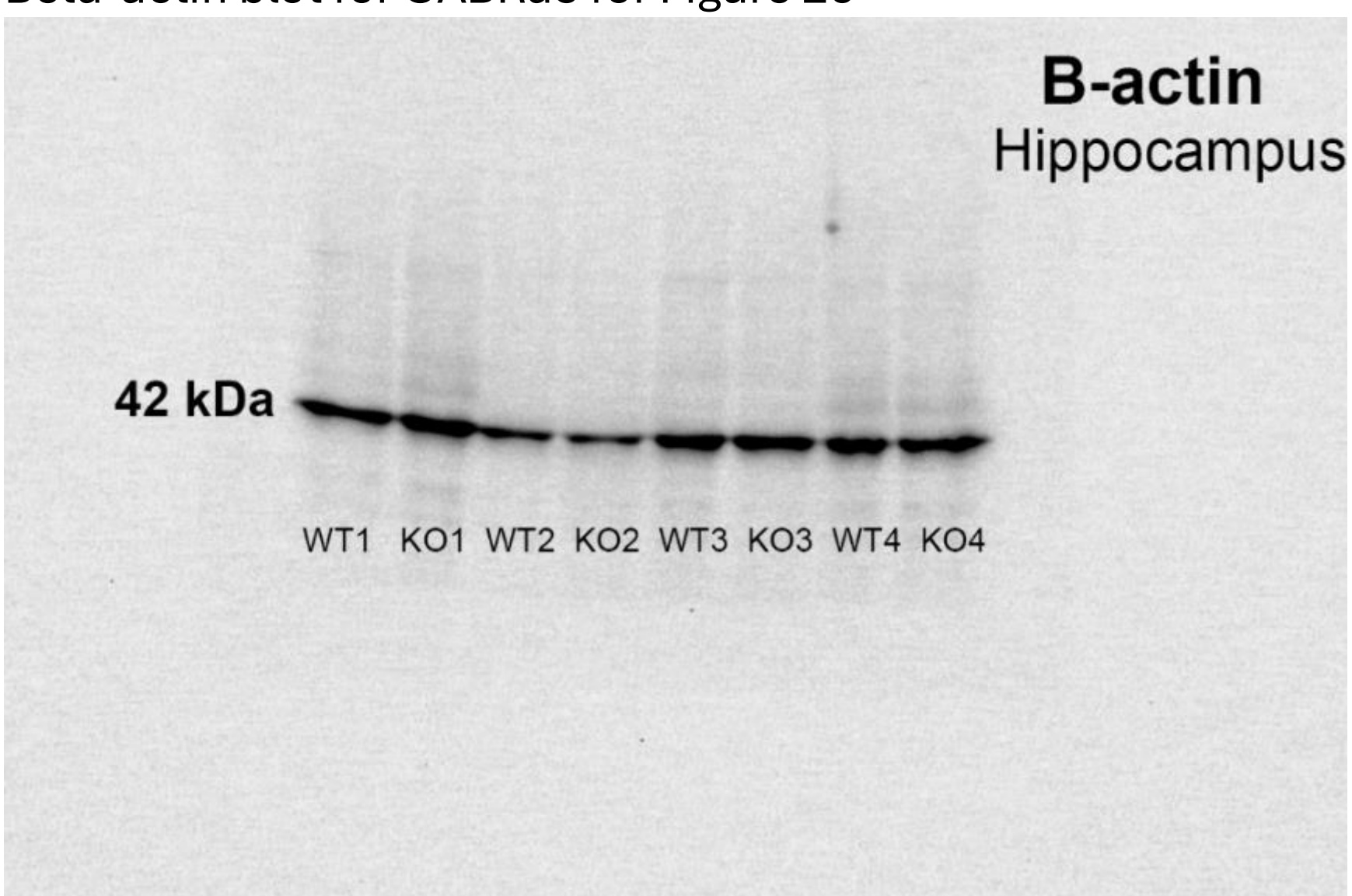

GAT3 blot for Figure 6d

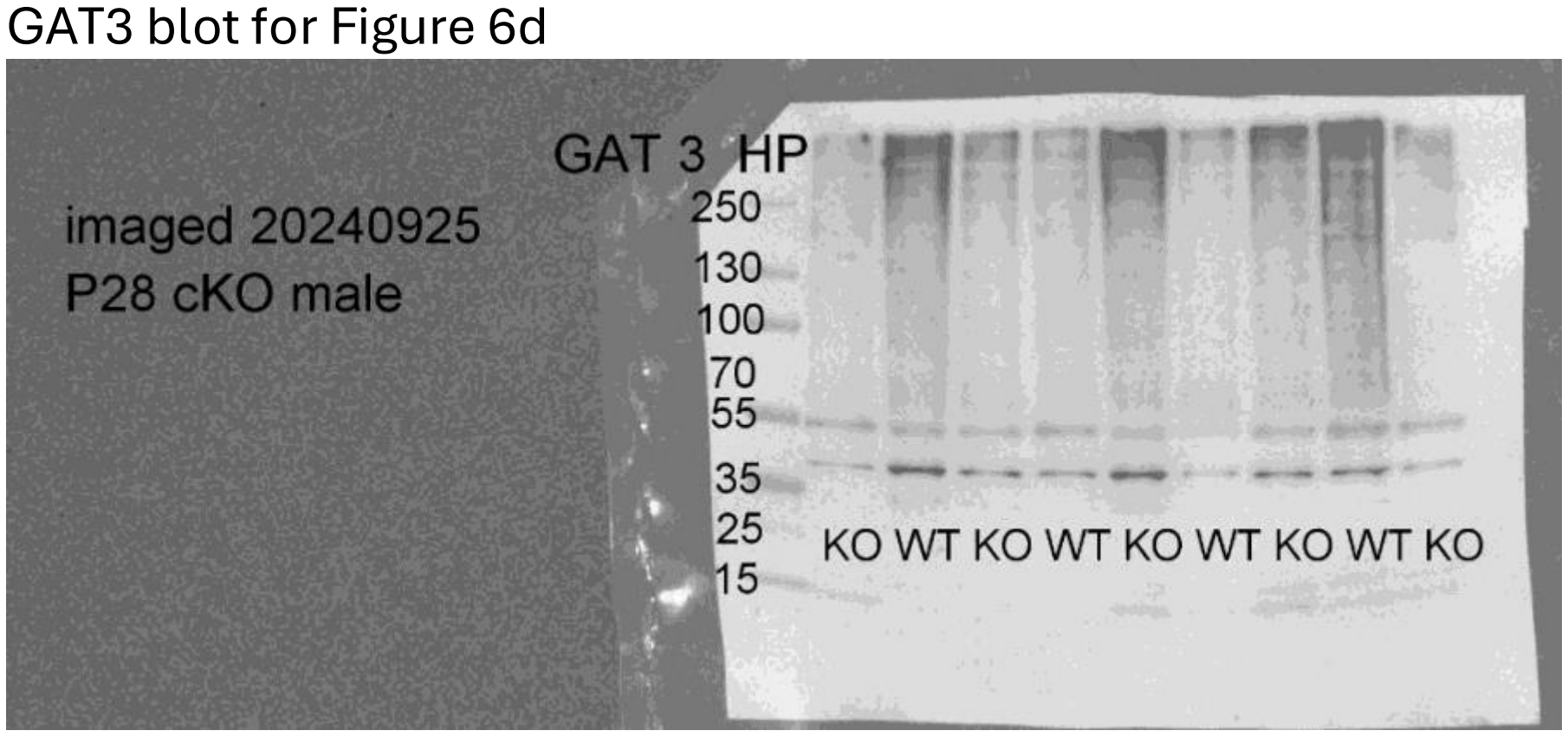

Beta-actin blot for GAT3 for Figure 6d

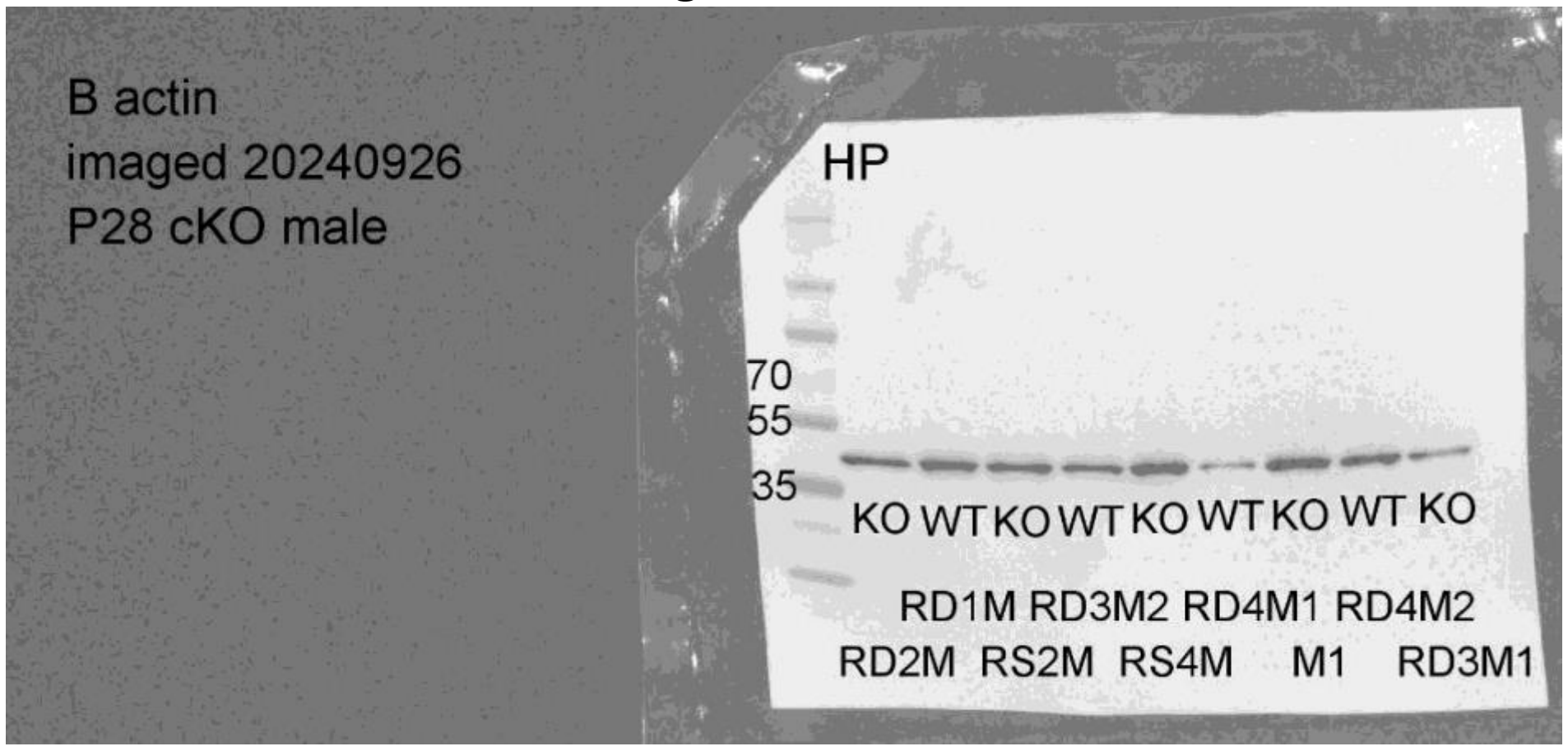

Supplement: Supplementary file 1 [file JNC-170-e70541-s001.pdf]
